# An IGFBP7^hi^ endothelial cell subset drives T cell extravasation in psoriasis via endothelial glycocalyx degradation

**DOI:** 10.1172/JCI160451

**Published:** 2023-05-01

**Authors:** Qingyang Li, Shuai Shao, Zhenlai Zhu, Jiaoling Chen, Junfeng Hao, Yaxing Bai, Bing Li, Erle Dang, Gang Wang

**Affiliations:** Department of Dermatology, Xijing Hospital, Fourth Military Medical University, Xi’an, Shaanxi, China.

**Keywords:** Inflammation, Vascular Biology, Endothelial cells, Skin

## Abstract

Dysfunction of vascular endothelial cells (ECs) facilitates imbalanced immune responses and tissue hyperinflammation. However, the heterogeneous functions of skin ECs and their underlying mechanism in dermatoses remain to be determined. Here, focusing on the pathogenic role of skin ECs in psoriasis, we characterized the molecular and functional heterogeneity of skin ECs from healthy individuals and psoriasis patients at the single-cell level. We found that endothelial glycocalyx destruction, a major feature of EC dysfunction in psoriasis, was a driving force during the process of T cell extravasation. Interestingly, we identified a skin EC subset, IGFBP7^hi^ ECs, in psoriasis. This subset actively responded to psoriatic-related cytokine signaling, secreted IGFBP7, damaged the endothelial glycocalyx, exposed the adhesion molecules underneath, and prepared the endothelium for immune-cell adhesion and transmigration, thus aggravating skin inflammation. More importantly, we provided evidence in a psoriasis-like mouse model that anti-IGFBP7 treatment showed promising therapeutic effects for restoring the endothelial glycocalyx and alleviating skin inflammation. Taken together, our results depict the distinct functions of EC clusters in healthy and psoriatic skin, identify IGFBP7^hi^ ECs as an active subset modulating vascular function and cutaneous inflammation, and indicate that targeting IGFBP7 is a potential therapeutic strategy in psoriasis.

## Graphical abstract

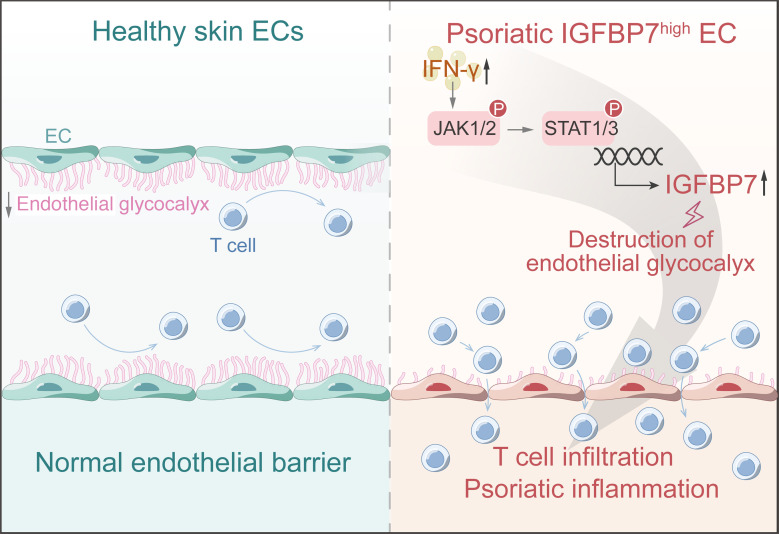

## Introduction

Blood vessels are vital life-supporting channels penetrating almost every organ and tissue in the human body. Vascular endothelial cells (ECs), the inner lining of blood vessels, not only form the endothelial barrier to maintain vascular integrity, but also actively regulate vessel function, orchestrate the local immune response, and participate in the pathogenesis of immune-mediated inflammatory diseases (1, 2). Increasing evidence demonstrates that ECs are not uniform cells, but rather have highly heterogeneous molecular and functional features along the vascular bed. Functional EC heterogeneity is particularly essential to regulating immune-cell adhesion and extravasation in many inflammatory settings, such as atherosclerosis, diabetes, and senescence (3, 4). Recent studies and our previous work have revealed heterogeneity within skin ECs, which are clustered into different subtypes with specific locations and functions (5, 6). However, the pathophysiologic role of endothelial heterogeneity in inflammatory dermatoses is currently unknown.

Diverse EC subsets together mediate the dynamic regulation of the endothelial barrier, which is necessary for pathological progression (7). The endothelial glycocalyx is a dynamic structure of proteoglycans and glycosaminoglycans covering the inner wall of blood vessels, and together with cellular junctions of ECs, it forms the endothelial barrier (8). Endothelial glycocalyx acts as the first line of defense in limiting the pathological entry of immune cells into the skin and maintaining a homeostatic milieu for proper skin function (9). Endothelial glycocalyx integrity is affected by various factors, including angiocrine factors secreted by ECs (10, 11), proteases and sheddases secreted by circulating cells (12), and mechanical and other chemical stresses within the bloodstream (13, 14). EC-derived angiocrine factors tightly modulate the dynamic equilibrium of endothelial glycocalyx degradation and reconstruction. For instance, IGFBP7, an angiocrine factor, can shed glycosaminoglycan fragments from the endothelial glycocalyx by directly binding to one of its major components, heparan sulfate (HS), which is distributed predominantly on the luminal side of the glycocalyx layer (15), thereby degrading the spatial structure of the endothelial glycocalyx and exposing the adhesion molecules underneath (16, 17). Overexpression of IGFBP7 in blood vessels increases vascular permeability and promotes tumor metastasis and liver fibrosis by facilitating cancer cell and Th17 cell extravasation, which has been reported in glioblastoma, melanoma, and nonalcoholic steatohepatitis (18–20). These studies suggest the critical role of the endothelial barrier in modulating inflammatory responses, with implications in inflammatory dermatoses.

To address how diverse skin EC subsets affect vascular function and immune responses, we focused on psoriasis, a common T cell–mediated inflammatory skin disease characterized by tortuous and dilated skin vessels. We constructed a single-cell transcriptional atlas of skin ECs and identified a group of IGFBP7^hi^ ECs in psoriatic skin vessels. We further showed that IGFBP7 promoted T cell adhesion to ECs by destroying the endothelial glycocalyx structure in psoriasis. Importantly, we highlighted the IGFBP7^hi^ EC subpopulation as a therapeutic target for inflammatory skin diseases. This study provides a comprehensive understanding of skin EC heterogeneity in psoriasis and demonstrates its pathogenic role in immune responses and cutaneous inflammation.

## Results

### The skin EC atlas of psoriasis patients and healthy controls.

**Figure 1:**
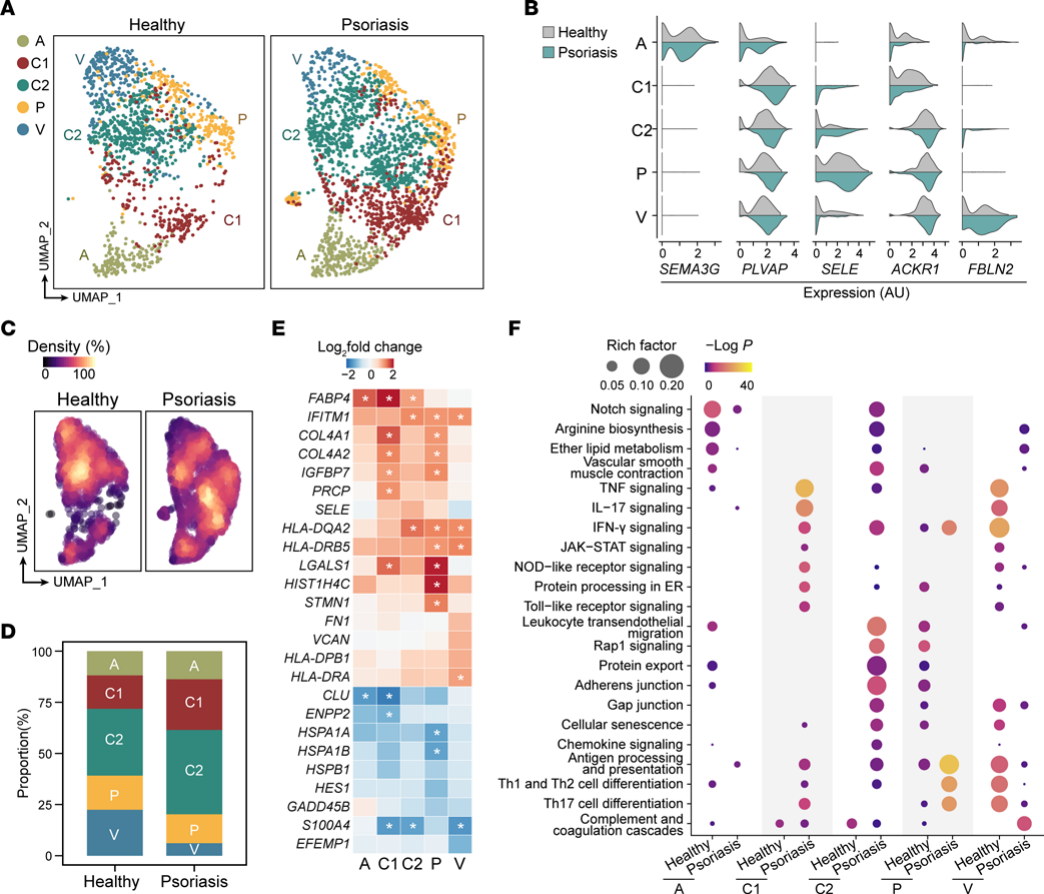
Skin EC landscape in psoriatic and healthy samples. (**A**) Uniform Manifold Approximation and Projection (UMAP) visualization showing 5 clusters of skin ECs in healthy and psoriatic scRNA-Seq data sets. Dots in different colors represent cells in corresponding clusters, which are cluster A (arteriole ECs); clusters C1, C2, and P (capillary ECs); and cluster V (venule ECs). (**B**) Violin plots showing the expression of discriminatory markers for each EC cluster from healthy and psoriatic skin. (**C**) Healthy and psoriatic ECs colored by cell density, with yellow indicating higher density and purple indicating lower density. (**D**) Bar charts showing the proportions of corresponding clusters in healthy and psoriatic skin ECs. (**E**) Heatmap showing the representative cluster-based DEGs between healthy and psoriatic ECs. Cells are colored by log_2_ fold change between the average expression of psoriatic EC clusters and healthy EC clusters. Asterisks represent log_2_ fold change of the average expression between psoriatic and healthy samples > 1 or < –1. (**F**) Functional enrichment analysis of cluster-based DEGs between healthy and psoriatic ECs. Color indicates the −log *P* value, and size indicates the rich factor.

To determine the molecular and functional heterogeneity of skin ECs in psoriasis, we isolated ECs from skin lesions of psoriasis patients (Supplemental Table 1; supplemental material available online with this article; https://doi.org/10.1172/JCI160451DS1) using magnetic- and fluorescence-activated cell sorting and further processed the samples for single-cell RNA–sequencing (scRNA-Seq). After the removal of low-quality cells and non-ECs, integration with the scRNA-Seq data set of healthy skin ECs published previously (6), and cell identity transfer (Supplemental Figure 1), a total of 1,424 healthy skin ECs and 2,121 psoriatic skin ECs were used for further analysis (National Genomics Data Center, https://ngdc.cncb.ac.cn, PRJCA007038 and PRJCA002692). Skin ECs were classified into 5 clusters: arteriole ECs (cluster A), capillary ECs (capillary ECs 1, cluster C1; capillary ECs 2, cluster C2; postcapillary venule ECs, cluster P), and venule ECs (cluster V) (Figure 1A). The expression of cluster signatures in psoriatic ECs was consistent with that in healthy skin ECs (Figure 1B).

We found that psoriatic ECs differed from healthy ECs in cluster composition, molecular features, and functional pathways. As shown by the cell-density plot (Figure 1C) and cluster proportion plot (Figure 1D), the proportion of capillary ECs (clusters C1 and C2) was increased in psoriasis, whereas venule ECs (cluster V) were diminished. In addition, differential gene expression analysis was performed on healthy and psoriatic ECs in each cluster (Supplemental Data File 1). Among all molecular alterations, key differentially expressed genes (DEGs) with a defined threshold (log_2_ fold change of the average expression between psoriasis and healthy ECs > 0.5 or **< −**0.5) and significant *P* value were highlighted (Figure 1E). Interestingly, various genes were upregulated in psoriatic capillary ECs (clusters C1, C2, and P), such as *IGFBP7*, *PRCP*, *HLA-DQA2*, and *LGALS1*. Functional enrichment analysis further revealed that these DEGs were involved in lipid metabolism, cytokine and chemokine signaling, endothelial barrier modulation, and interactions with immune cells (Figure 1F). Notably, capillary ECs exhibited a series of functional alterations. For instance, TNF-α–, IL-17–, IFN-γ–, and JAK/STAT-signaling pathways were enriched in psoriatic cluster C1 cells. Leukocyte transendothelial migration was enriched in psoriatic cluster C2. Antigen presentation and Th17 cell differentiation were enriched in psoriatic cluster P (Figure 1F). Psoriatic capillary ECs in different clusters cooperatively fine-tune the recruitment, adhesion, migration, and activation of immune cells. Together, the increased abundance and substantial functional alterations in psoriatic capillary ECs indicate the importance of capillary ECs in psoriasis pathogenesis.

### The endothelial glycocalyx is disrupted in psoriasis and correlated with disease severity.

**Figure 2:**
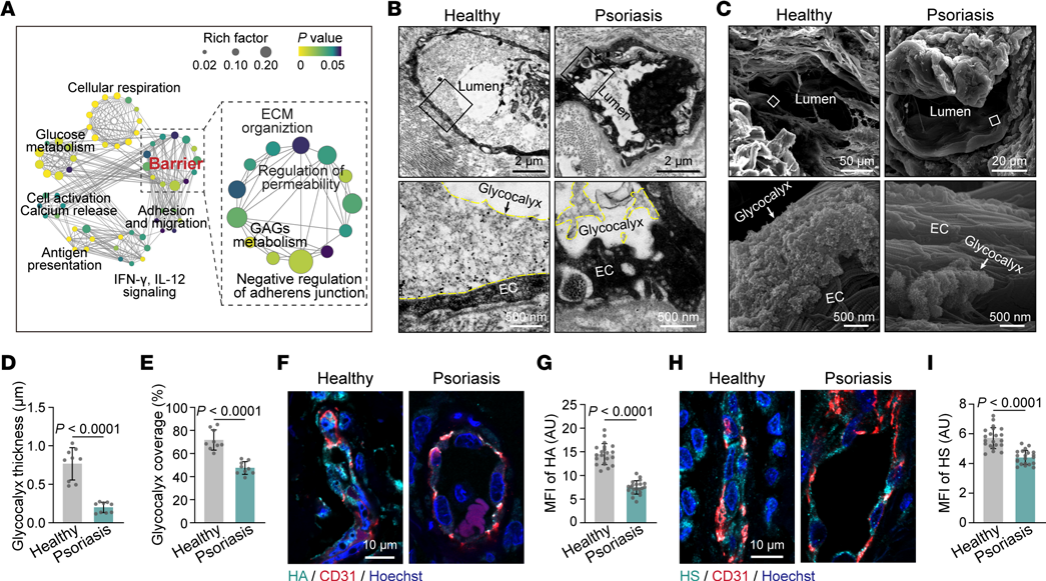
The endothelial glycocalyx is disrupted in psoriatic skin vessels. (**A**) Network visualization of pathways enriched in psoriatic capillary ECs compared with healthy capillary ECs. Network nodes, which are colored by adjusted *P* value and sized by rich factor, represent individual enriched gene sets; edges represent shared genes between nodes. (**B** and **C**) TEM and SEM showing the endothelial glycocalyx in skin blood vessels from healthy individuals and psoriasis patients (*n* = 3 skin samples/group). The endothelial glycocalyx is highlighted by the yellow dotted line. (**D** and **E**) Average endothelial glycocalyx thickness and coverage in skin vessels of healthy individuals and psoriasis patients (*n* = 9 vessels of 3 skin samples/group). (**F** and **G**) Immunofluorescence and MFI quantification of HA on skin ECs from healthy subjects and psoriasis patients (*n* = 21 vessels of 7 skin samples/group). (**H** and **I**) Immunofluorescence and MFI quantification of HS on skin ECs from healthy subjects and psoriasis patients (*n* = 21 vessels of 7 skin samples/group). Data are represented as mean ± SD. Analysis was performed using unpaired Student’s *t* test.

Next, the most varied capillary ECs (clusters C1, C2, and P) of healthy individuals and psoriasis patients were extracted from the scRNA-Seq data sets for further analysis. Functional enrichment analysis of DEGs in psoriatic capillary ECs showed that, apart from immune-related pathways, genes related to the endothelial barrier were highly activated, including glycosaminoglycan metabolism and adherens junction (Figure 2A). Gene set enrichment analysis (GSEA) showed that protein glycosylation pathways involved in endothelial glycocalyx metabolism were significantly enriched in psoriatic capillary ECs (Supplemental Figure 2A). Moreover, endothelial glycocalyx–deteriorating genes related to angiocrine factors (e.g., *IGFBP7*, *HYAL2*, and *BSG*), shear stress (e.g., *KLF2*), and ROS (e.g., *APEX1*) (21–25) were overexpressed in psoriatic capillary ECs compared with healthy capillary ECs (Supplemental Figure 2B). Therefore, these data suggest that endothelial glycocalyx destruction could be a major characteristic of psoriatic capillary ECs.

We further explored whether the endothelial glycocalyx was disrupted in psoriasis at the ultrastructural level. Transmission electron microscopy (TEM) analysis revealed a bush-like layer that covered ECs on the luminal side in healthy skin vessels, whereas the endothelial glycocalyx structure was thinner and discontinuous in psoriatic skin vessels (Figure 2B). Scanning electron microscopy (SEM) analysis also confirmed this phenomenon (Figure 2C) and showed that the endothelial glycocalyx formed moss-like structures covering the endothelium of healthy skin vessels, whereas the endothelial glycocalyx was reduced and shattered in psoriatic skin vessels. Quantitative analysis further demonstrated that the thickness and coverage of the endothelial glycocalyx in psoriatic skin blood vessels were significantly less than those in healthy skin vessels (Figure 2, D and E). Hyaluronic acid (HA) and HS are the major glycosaminoglycans constituting the endothelial glycocalyx, and circulating HA and HS levels could partially reflect endothelial glycocalyx degradation in clinical assessment (26). Immunofluorescence of skin tissues from psoriasis patients showed a significantly reduced MFI of HA and HS in blood vessels compared with that in healthy individuals (Figure 2, F–I). Correspondingly, the serum levels of HA and HS were increased in the psoriasis patients, and the serum level of HS was positively correlated with the disease severity of psoriasis (Supplemental Figure 2, C–E). These data demonstrate that the endothelial glycocalyx of the skin vasculature is degraded in psoriasis.

### Endothelial glycocalyx degradation drives T cell extravasation into skin lesions.

**Figure 3:**
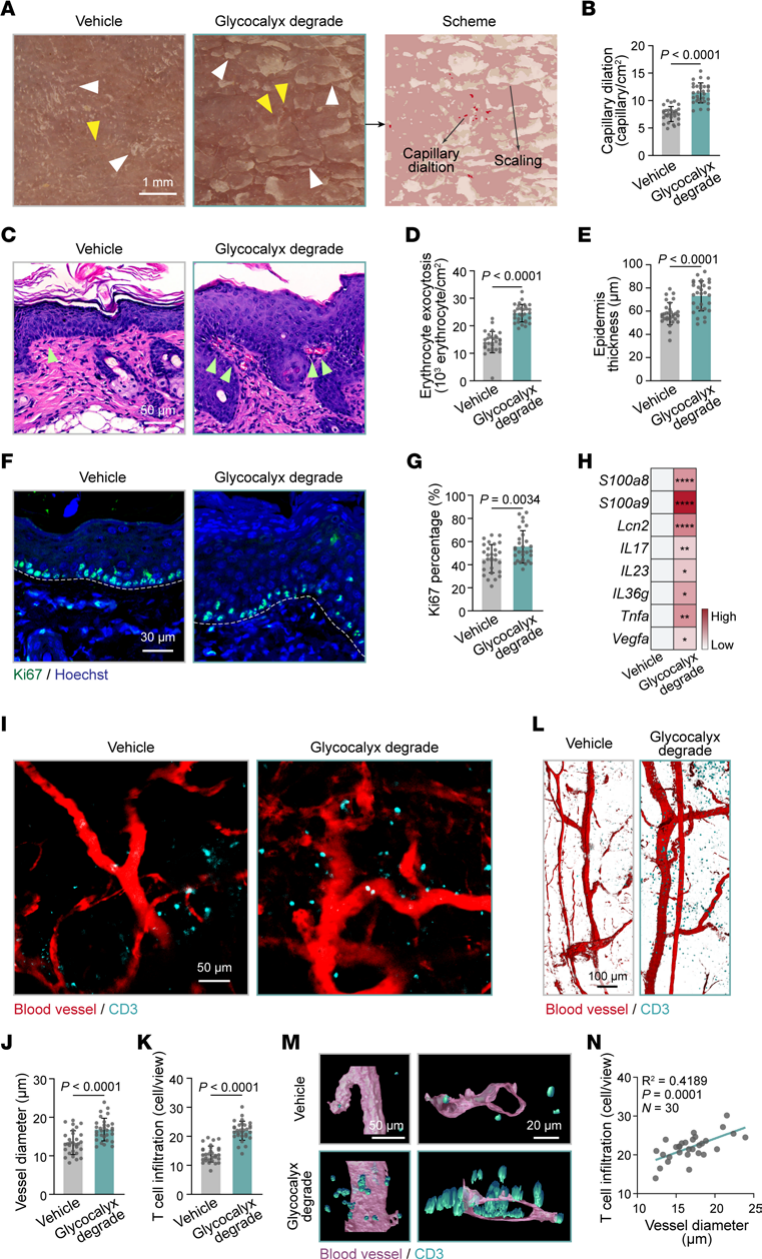
Degradation of the endothelial glycocalyx aggravates skin inflammation in psoriasis. (**A**) Dermatoscopy of dorsal skin in IMQ mice (*n* = 6 mice/group) with or without endothelial glycocalyx degradation. Yellow arrowheads, pointing at red dots, indicate dilated skin capillaries; white arrowheads, pointing at yellowish plates, indicate psoriatic scales. A dermatoscopic schematic is shown on the right. (**B**) Quantification of dilated capillaries in **A** (*n* = 30 views/group). (**C**) H&E staining of dorsal skin from IMQ mice (*n* = 6 mice/group) with or without endothelial glycocalyx degradation. Green arrowheads indicate erythrocyte exocytosis. (**D** and **E**) Quantification of erythrocyte exocytosis and epidermis thickness in **C** (*n* = 30 views/group). (**F**) Immunofluorescence for Ki67 (green) and Hoechst (blue) in dorsal skin from IMQ mice (*n* = 6 mice/group) with or without endothelial glycocalyx degradation. The dashed white lines mark the interface between the epidermis and dermis. (**G**) Quantification of the percentage of Ki67^+^ cells in the basal layer in **F** (*n* = 30 views/group). (**H**) Heatmap showing the transcriptional levels of inflammatory genes in the skin of IMQ mice with or without endothelial glycocalyx degradation (*n* = 6 mice/group). (**I**) Whole-mount immunofluorescence staining for blood vessels (red) and CD3 (cyan) in ear skin from IMQ mice (*n* = 6 mice/group) with or without endothelial glycocalyx degradation. (**J** and **K**) The average blood vessel diameter and infiltrated T cells show in **I** were quantified (*n* = 30 views/group). (**L** and **M**) 3D surface rendering of blood vessels and CD3 based on whole-mount immunofluorescence staining. (**N**) Linear regression analysis of the correlation between the average vessel diameter and the number of infiltrated T cells per view in IMQ mice with endothelial glycocalyx degradation. Data are represented as mean ± SD. Analysis was performed using unpaired Student’s *t* test. *P < 0.05; **P < 0.01; ****P < 0.0001.

To explore the regulatory role of the endothelial glycocalyx in skin inflammation, we then i.v. injected hyaluronidase and heparinase III (27, 28) into mice to break down HA and HS, respectively, followed by imiquimod (IMQ) induction to establish a robust psoriasis-like mouse model (29). TEM confirmed that the endothelial glycocalyx layer was degraded in the dorsal skin of control mice after treatment with hyaluronidase and heparinase III (Supplemental Figure 3). Under dermatoscopy, increased red dots, representing dilated capillaries in the papillary dermis, and patchy yellowish scales, representing abnormal epidermal proliferation, were observed on the dorsal skin of IMQ-induced psoriasis-like mice (IMQ mice) with endothelial glycocalyx degradation compared with IMQ-alone mice (Figure 3, A and B, and Supplemental Figure 4, A and B). Histological examinations showed that erythrocyte exocytosis, epidermal thickness, and the percentage of Ki67^+^ cells within the basal layer were significantly increased in endothelial glycocalyx–degraded IMQ mice (Figure 3, C–G, and Supplemental Figure 4, C–G). In addition, the mRNA levels of psoriasis-related cytokines and antimicrobial peptides were higher in endothelial glycocalyx–degraded IMQ mice than in IMQ-alone mice (Figure 3H and Supplemental Figure 4H). These data suggest that endothelial glycocalyx degradation aggravates psoriasis-like dermatitis.

Given the key role of endothelial glycocalyx integrity in controlling immune-cell extravasation, we further detected the skin vasculature and T cell infiltrates in mice with a psoriasis-like phenotype. Immunofluorescence images showed dilated and tortuous cutaneous blood vessels (marked by 500 kDa tetramethylrhodamine isothiocyanate–dextran [TRITC-dextran]) in the IMQ mice, which were further exacerbated by endothelial glycocalyx degradation (Figure 3, I and J, and Supplemental Figure 4, I–K). Additionally, perivascular leakage of dextran was observed in endothelial glycocalyx–degraded IMQ mice (Figure 3I), suggesting that endothelial glycocalyx destruction induced barrier hyperpermeability and vascular dysfunction. Notably, infiltrated CD3^+^ T cells (marked by cyan) in the IMQ mice were significantly increased after endothelial glycocalyx degradation and were closely situated along the dilated skin vessels (Figure 3, I and K, and Supplemental Figure 4, L–N). A 3D reconstruction of the immunofluorescence staining further depicted increased CD3^+^ T cells inside, across, and outside the blood vessels (Figure 3, L and M), indicating an intimate spatial relationship between infiltrated T cells and skin vessels. The number of infiltrated T cells was positively correlated with blood vessel diameter (Figure 3N). Taken together, these in vivo data indicate that degradation of the endothelial glycocalyx leads to an impaired endothelial barrier and facilitates T cell extravasation, thereby aggravating cutaneous inflammation.

### The IGFBP7^hi^ EC subset is expanded in psoriatic skin lesions.

**Figure 4:**
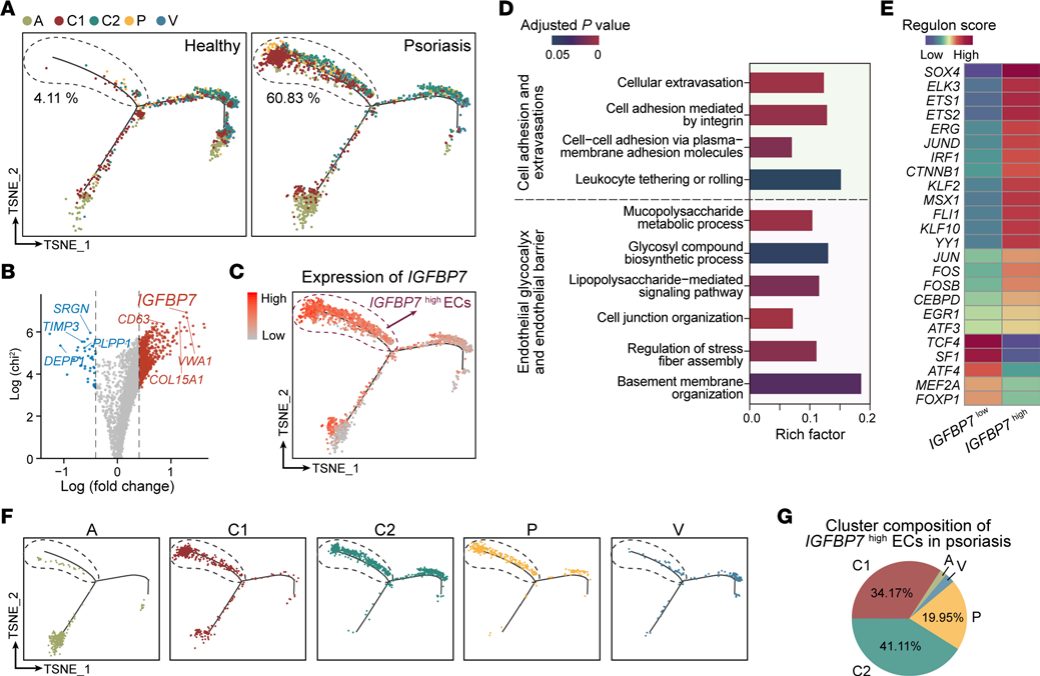
IGFBP7^hi^ ECs are increased in psoriatic skin. (**A**) Pseudotime trajectory of healthy and psoriatic ECs. (**B**) Volcano plot of DEGs between the psoriatic ECs (circled by dashed line) and the rest of the psoriatic ECs (see **A** right panel). Genes with log (fold change) > 0 are upregulated in psoriatic ECs (circled by dashed line). (**C**) *IGFBP7* expression in psoriatic ECs. (**D**) GO analysis showing the pathways enriched in psoriatic *IGFBP7*^hi^ ECs compared with psoriatic *IGFBP7*^lo^ ECs. The *x* axis represents the rich factor. Bar colors indicate adjusted *P* value. (**E**) Regulon activity of *IGFBP7*^hi^ and *IGFBP7*^lo^ ECs. (**F**) Pseudotime trajectory of each cluster from psoriatic ECs. (**G**) Cluster composition of psoriatic *IGFBP7*^hi^ ECs.

To identify the key EC subset mediating endothelial dysfunction and glycocalyx destruction in psoriasis, we applied pseudotime analysis of healthy and psoriatic EC scRNA-Seq data sets. As the pseudotime trajectory showed (Figure 4A), there were 2 diverging cell states (state 1 on the left branch and state 2 on the right branch), both of which started at cluster A and progressed toward clusters C1, C2, P, and V, indicating arteriovenous zonation in skin ECs, as we have discovered previously (6). Interestingly, 60.83% of psoriatic skin ECs diverged to the left branch, while only 4.11% of healthy skin ECs progressed along the left branch (Figure 4A), indicating that this subset of ECs was distinctly activated in psoriasis. Next, to characterize this unique EC subset, we compared the gene expression between psoriatic ECs on the left branch and the other psoriatic ECs (Supplemental Data File 2). Among all the upregulated genes, *IGFBP7* ranked as the most significant (Figure 4B). The expression of *IGFBP7* successfully discriminated the 2 populations of psoriatic skin ECs within the pseudotime trajectory, that is, the *IGFBP7*^hi^ (on the left branch) and *IGFBP7*^lo^ ECs (the rest) (Figure 4C and Supplemental Figure 5). Gene Ontology (GO) pathway analysis revealed that cell adhesion and extravasation, endothelial glycocalyx, and endothelial barrier were enriched in psoriatic *IGFBP7*^hi^ ECs compared with psoriatic *IGFBP7*^lo^ ECs (Figure 4D). Single-cell regulatory network inference and clustering (SCENIC) analysis revealed 24 regulons (transcription factors and coexpressed target genes) enriched in psoriatic *IGFBP7*^hi^ ECs compared with psoriatic *IGFBP7*^lo^ ECs (Figure 4E), including *SOX4*, *ELK3*, and *ETS1*, which are critical for transendothelial infiltration (30, 31). These transcription factors may underpin the branch point at which psoriatic *IGFBP7*^hi^ and *IGFBP7*^lo^ ECs diverged, leading to a diverse functional state of skin ECs.

**Figure 5:**
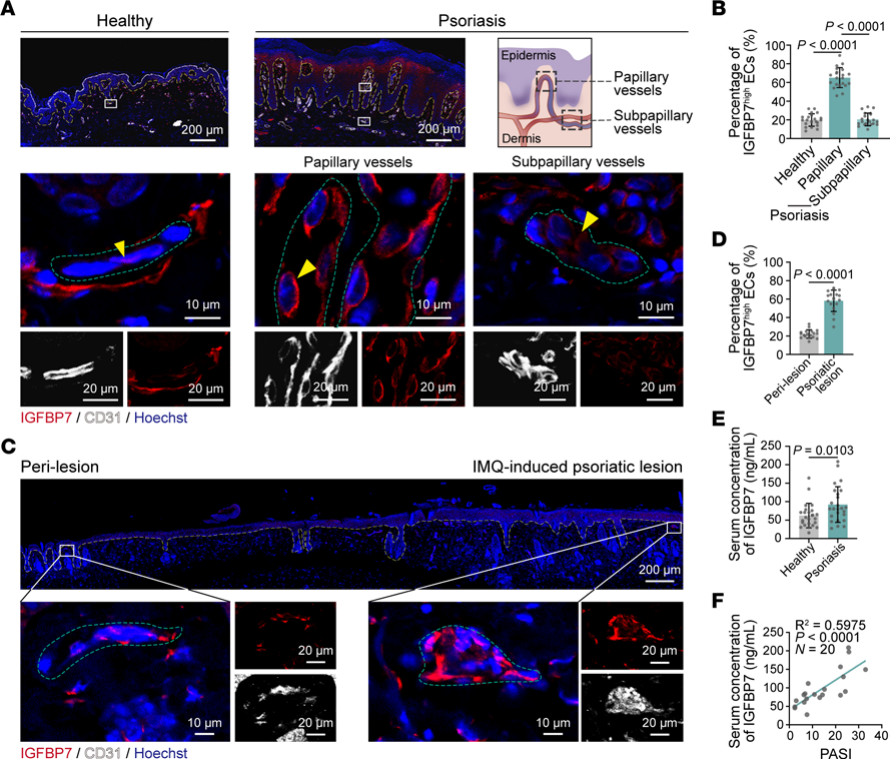
IGFBP7^hi^ ECs accumulate in papillary vessels of psoriasis skin. (**A**) Immunofluorescence staining of IGFBP7, CD31, and Hoechst in skin tissues from healthy individuals and psoriasis patients (*n* = 7 skin samples/group). The yellow dashed lines mark the interface between the epidermis and dermis. The green dashed lines indicate the outline of ECs. The yellow arrowheads indicate skin ECs that express IGFBP7. Schematic of papillary and subpapillary vessels in the psoriatic skin lesion is shown on the right. (**B**) The percentages of IGFBP7^hi^ ECs in blood vessels of healthy and psoriatic skin in **A** were quantified (*n* = 21 views of 7 skin samples/group). (**C**) Immunofluorescence staining for IGFBP7, CD31, and Hoechst in IMQ-induced psoriatic skin lesions and perilesions (*n* = 6 mice). The yellow dashed lines mark the interface between the epidermis and dermis. The green dashed lines indicate the outline of ECs. (**D**) Percentages of IGFBP7^hi^ ECs in blood vessels were quantified (*n* = 18 views of 6 skin samples/group). (**E**) IGFBP7 levels in peripheral blood from patients (*n* = 20 blood samples/group). (**F**) Linear regression analysis of the correlation between serum IGFBP7 and the psoriasis area and severity index (PASI) in psoriasis patients. Data are represented as mean ± SD. Analysis of data in **B** was performed using 1-way ANOVA with Tukey’s post hoc test. Data in **D** and **E** were analyzed using unpaired Student’s *t* test.

We then questioned which blood vessel taxonomy (arterioles, capillaries, and venules) *IGFBP7*^hi^ ECs belonged to. As shown in the pie chart, psoriatic *IGFBP7*^hi^ ECs were predominantly composed of capillary ECs (clusters C1, C2, and P) (Figure 4, F and G). These capillary ECs were mainly located in the superficial and intermediate plexuses, which were in the upper dermis, based on our previous study (6). We thus determined whether IGFBP7^hi^ ECs existed and their exact histological location in psoriatic skin lesions. Tissue immunofluorescence of skin lesions from psoriasis patients confirmed the presence of IGFBP7^hi^ ECs, which were markedly increased compared with those in healthy individuals (Figure 5A). To our surprise, we discovered an interesting distribution pattern of IGFBP7^hi^ ECs. Taking the lower line of rete pegs as a separating line, blood vessels above this line, which were within the dermal papillae, were defined as papillary vessels, whereas blood vessels below this line were defined as subpapillary vessels (Figure 5A). In psoriatic skin lesions, IGFBP7^hi^ ECs were mainly located in the papillary vessels, whereas IGFBP7^lo^ ECs were located in the subpapillary vessels (Figure 5B). In addition, the existence and distribution of IGFBP7^hi^ ECs were also examined in IMQ mice via immunofluorescence costaining, which showed that the percentages of IGFBP7^hi^ ECs were higher in lesional skin than in perilesional skin and normal control skin (Figure 5, C and D, and Supplemental Figure 6). IGFBP7 exerts endothelial glycocalyx–degrading effects mainly in its secreted form by binding to HS, the main endothelial glycocalyx component (16, 17). Our ELISA results showed a significant elevation of serum IGFBP7 in psoriasis patients, which was positively correlated with disease severity (Figure 5, E and F). These results suggest that the IGFBP7^hi^ EC subpopulation modulates the endothelial glycocalyx in a paracrine manner, and IGFBP7 may be the defining angiocrine factor in psoriasis.

### Elevated IGFBP7 drives endothelial dysfunction and skin inflammation.

**Figure 6:**
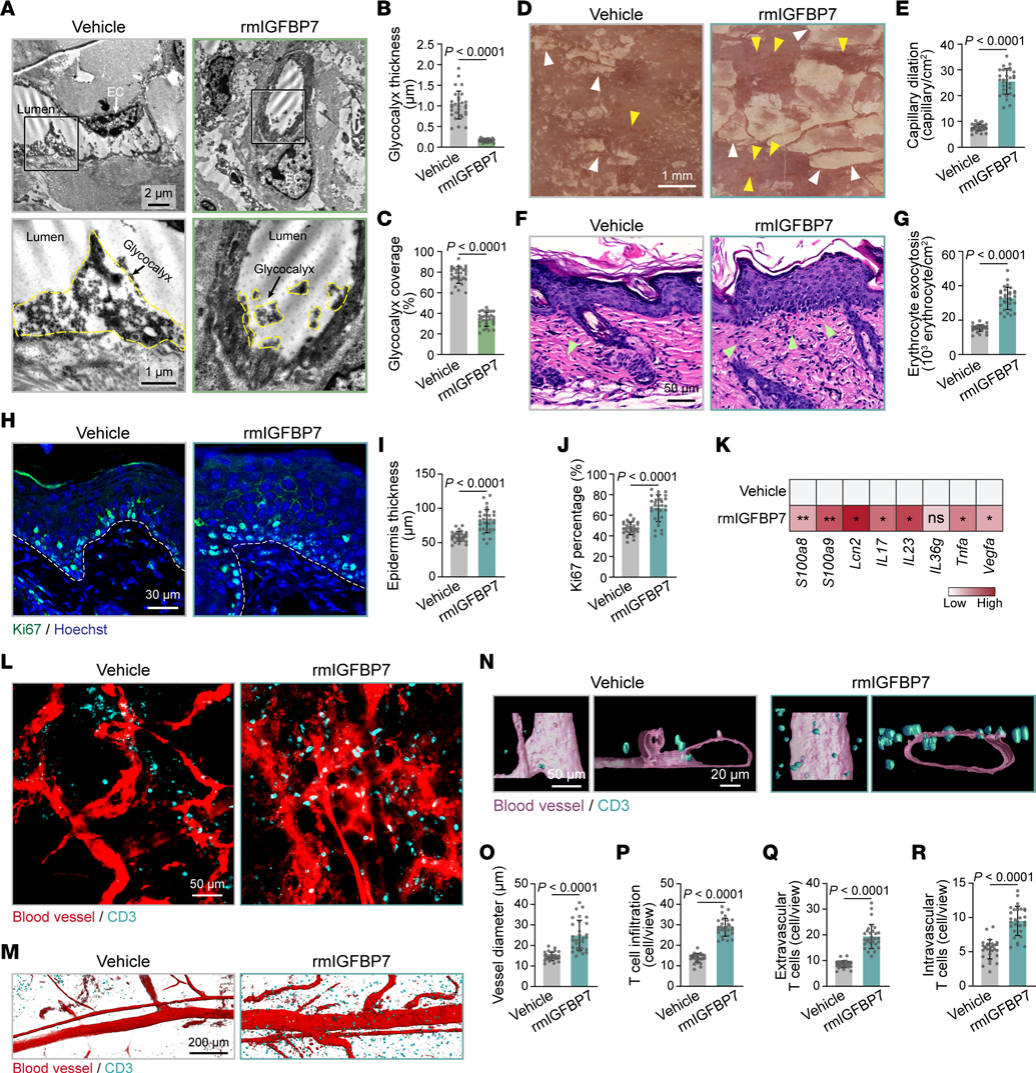
Elevated IGFBP7 leads to endothelial dysfunction and skin inflammation in IMQ mice. (**A**) TEM of skin endothelial glycocalyx in control mice (*n* = 6 mice/group) that received rmIGFBP7 or vehicle i.v. The endothelial glycocalyx is highlighted by the yellow dotted lines. (**B** and **C**) Endothelial glycocalyx thickness and coverage were quantified (*n* = 30 vessels of 6 mice/group). (**D**) Dermatoscopy of IMQ mice (*n* = 6 mice/group) with or without rmIGFBP7 injection. Yellow arrowheads indicate dilated skin capillaries; white arrowheads indicate psoriatic scales. (**E**) Quantification of dilated capillaries in **D** (*n* = 30 views of 6 mice/group). (**F**) Skin histology of IMQ mice (*n* = 6 mice/group) treated with or without rmIGFBP7. Green arrowheads indicate erythrocyte exocytosis. (**G**) Quantification of erythrocyte exocytosis in **F** (*n* = 30 views of 6 mice/group). (**H**) Immunofluorescence staining for Ki67 (green) in dorsal skin from IMQ mice (*n* = 6 mice/group) with or without rmIGFBP7 injection. Dashed white lines mark the interface between the epidermis and dermis. (**I** and **J**) Epidermis thickness and the percentage of Ki67^+^ cells in the basal layer in **H** were quantified (*n* = 30 views of 6 mice/group). (**K**) Inflammatory gene expression in skin tissues from IMQ mice treated with or without rmIGFBP7 (*n* = 6 mice/group). (**L**) Whole-mount immunofluorescence staining for skin blood vessels (red) and CD3 (cyan) in IMQ mice (*n* = 6 mice/group) treated with or without rmIGFBP7. (**M** and **N**) 3D surface rendering of blood vessels and CD3 based on whole-mount immunofluorescence staining. (**O**–**R**) Quantifications of blood vessel diameter, infiltrated T cells, extravascular T cells, and intravascular T cells (*n* = 30 views of 6 mice/group). Data are represented as mean ± SD. Significance was calculated using unpaired Student’s *t* test. *P < 0.05; **P < 0.01.

To investigate how secreted IGFBP7 influenced the endothelium and participated in psoriatic skin inflammation in vivo, mice were i.v. injected with recombinant murine IGFBP7 (rmIGFBP7). As expected, the endothelial glycocalyx layer was degraded in the dorsal skin of mice by i.v. injection of rmIGFBP7 (Figure 6, A–C). In IMQ mice, rmIGFBP7 significantly aggravated psoriatic skin scaling (Figure 6D and Supplemental Figure 7A), skin capillary dilation (Figure 6E and Supplemental Figure 7B), erythrocyte exocytosis, epidermal proliferation (Figure 6, F–J, and Supplemental Figure 7, C–G), and inflammatory responses (Figure 6K and Supplemental Figure 7H). These data show that IGFBP7 exacerbates psoriasis-like skin inflammation.

Cutaneous vessels were further visualized by whole-mount immunofluorescence staining, which showed that rmIGFBP7 treatment in control mice led to dilated and tortuous skin vessels (Supplemental Figure 7, I–K), similar to the vessel morphology in skin lesions of psoriasis patients. In IMQ mice, rmIGFBP7 treatment not only significantly increased vessel diameter, but also induced evident leakage of TRITC-dextran outside the vessels in IMQ mice (Figure 6L), indicating damaged barrier function. Moreover, CD3^+^ T cell infiltration was significantly increased and closely located along the skin blood vessels in rmIGFBP7-treated IMQ mice compared with IMQ-alone mice, as shown by whole-mount immunofluorescence and 3D reconstruction (Figure 6, L–R, and Supplemental Figure 7, L–N). Taken together, our data indicate that IGFBP7 deteriorates the endothelial glycocalyx, dampens endothelial barrier function, and increases T cell adhesion and infiltration in skin lesions, thus aggravating psoriatic inflammation.

### IGFBP7 enhances T cell adhesion by deteriorating the endothelial glycocalyx.

**Figure 7:**
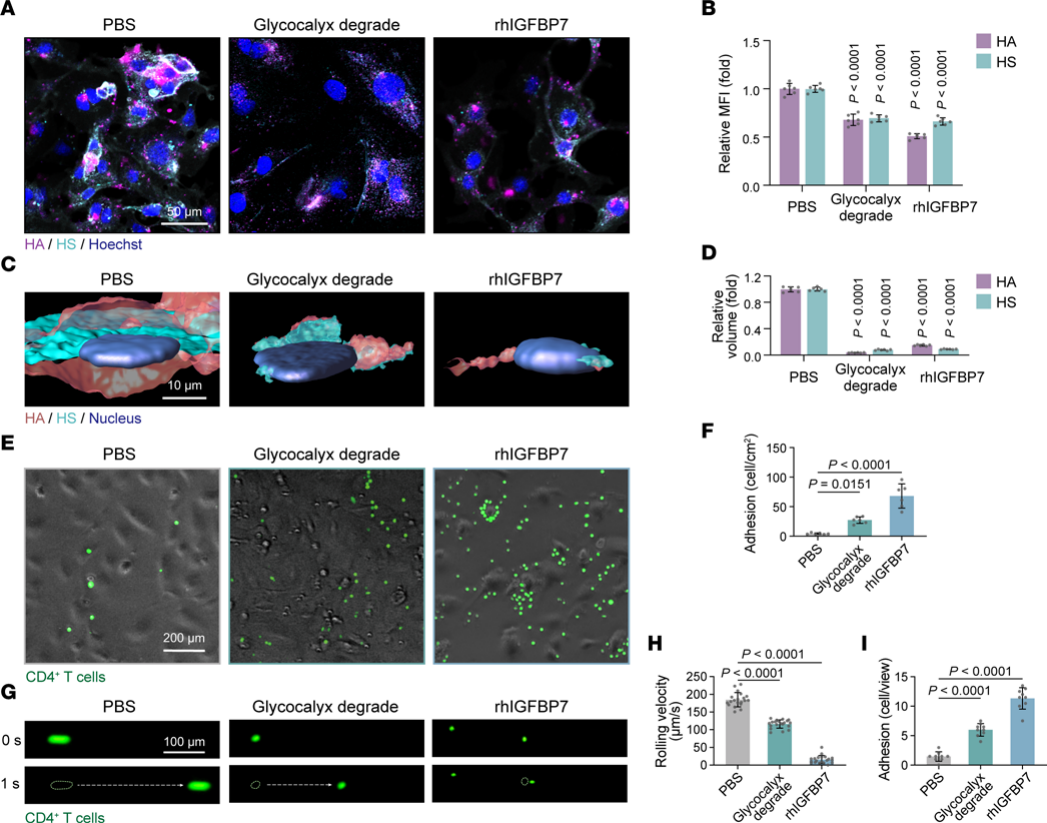
IGFBP7 induces endothelial glycocalyx degradation and promotes T cell adhesion. (**A** and **B**) Representative staining and MFIs of HA and HS in HMEC-1 cells (*n* = 6/group) treated with PBS, endothelial glycocalyx degradation, or rhIGFBP7. (**C** and **D**) 3D reconstruction and quantification of HA and HS volume (*n* = 6/group). (**E** and **F**) Representative images and quantification of adherent CD4^+^ T cells after coculturing with HMEC-1 cells, which were pretreated with PBS, endothelial glycocalyx degradation, or rhIGFBP7 (*n* = 6/group). (**G**) Snapshots of CD4^+^ T cells flowing on HMEC-1 cells at different time points (*n* = 6/group). (**H**) The rolling velocity of CD4^+^ T cells in **G** (*n* = 20 T cells/group). (**I**) The number of adherent CD4^+^ T cells (*n* = 9 views/group) at the end of the flow assay in **G**. Data are represented as mean ± SD. All data were analyzed using 1-way ANOVA with Tukey’s post hoc test. Labeled *P* values in **B** and **D** represent the differences between the corresponding group and the PBS group.

To probe the modulatory effects of secreted IGFBP7 on the endothelial glycocalyx and cell adhesion in vitro, we treated the human dermal microvascular EC line HMEC-1 with recombinant human IGFBP7 (rhIGFBP7) for 3 hours. Treatment with hyaluronidase and heparinase III for 3 hours was used as the positive control to degrade the endothelial glycocalyx. Immunofluorescence staining and 3D reconstruction showed that rhIGFBP7 reduced the MFI and volume of HA and HS (Figure 7, A–D).

To examine the impact of secreted IGFBP7 on cell adhesion, HMEC-1 cells pretreated with rhIGFBP7 or with degraded endothelial glycocalyx were cocultured with human CD4^+^ T cells. After 5 hours of static coculturing, CD4^+^ T cells that did not adhere to HMEC-1 cells were washed off. The number of T cells adhered to HMEC-1 cells was 13-fold higher in the rhIGFBP7-treated group than in the control group and was higher than that in the endothelial glycocalyx–degraded group (Figure 7, E and F). In addition, a flow assay was carried out to imitate the in vivo flowing, rolling, and adhesion of T cells in blood vessels. HMEC-1 cells grown on the bottom of a flow chamber were treated with rhIGFBP7 or hyaluronidase and heparinase III to degrade the endothelial glycocalyx. Then a flush of human CD4^+^ T cells was pumped into the chamber. Similarly to what occurred in the endothelial glycocalyx–degraded group, rhIGFBP7 substantially reduced the rolling velocity of CD4^+^ T cells on HMEC-1 cells (Figure 7, G and H), even leading to firm adhesion (Figure 7G), and eventually, more T cells adhered (Figure 7I and Supplemental Videos 1–3). Notably, neither rhIGFBP7 treatment nor degradation of the endothelial glycocalyx influenced the expression of adhesion molecules in HMEC-1 cells (Supplemental Figure 8). Given that the endothelial glycocalyx on the endothelium normally shields adhesion molecules underneath and restrains immune-cell adhesion (9, 32), we hypothesize that secreted IGFBP7 promotes T cell adhesion and infiltration mainly by binding to HS, shedding the endothelial glycocalyx, and exposing adhesion molecules.

### IFN-γ upregulates IGFBP7 in ECs and promotes T cell adhesion via the JAK1/2-STAT1/3 pathway.

**Figure 8:**
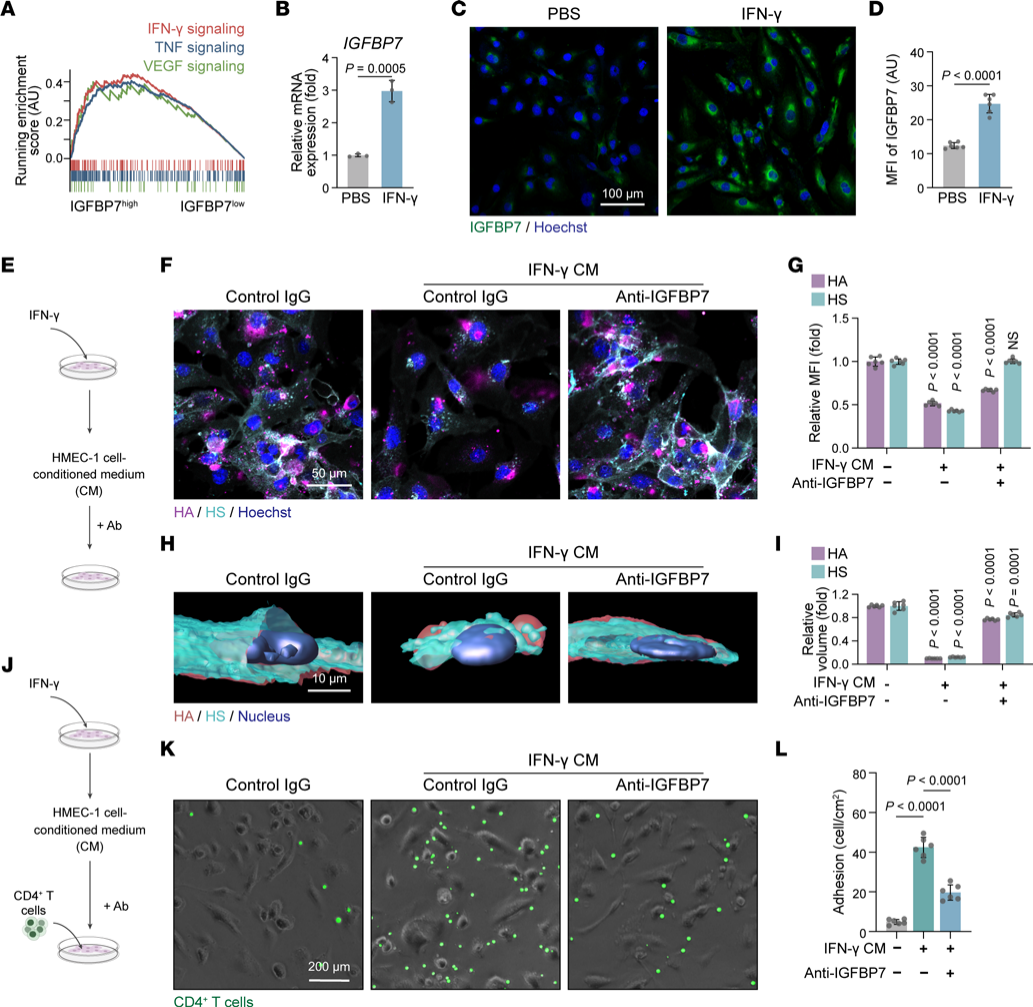
Anti-IGFBP7 treatment rescues IFN-γ–induced endothelial glycocalyx destruction and T cell adhesion. (**A**) GSEA revealing the signaling pathways enriched in IGFBP7^hi^ psoriatic ECs compared with IGFBP7^lo^ psoriatic ECs. (**B**) Relative mRNA expression assessed by quantitative real-time PCR of *IGFBP7* in HMEC-1 cells (*n* = 3/group) treated with PBS or IFN-γ. (**C** and **D**) Representative staining and MFI quantification of IGFBP7 in HMEC-1 cells (*n* = 6/group) treated with PBS or IFN-γ. (**E**) HMEC-1 cells stimulated with different conditioned media (CM) were treated with anti-IGFBP7 or control IgG. (**F** and **G**) Representative staining and MFI quantification of HA and HS (*n* = 6/group). (**H** and **I**) 3D reconstruction and volume quantification of HA and HS (*n* = 6/group). (**J**) HMEC-1 cells stimulated with different conditioned media were first treated with anti-IGFBP7 or control IgG and then cocultured with CD4^+^ T cells. (**K** and **L**) Representative images and quantification of adherent CD4^+^ T cells in the coculture assay (*n* = 6/group). Data are represented as mean ± SD. Data in **B** and **D** were analyzed using unpaired Student’s *t* test. Data in **G**, **I**, and **L** were analyzed using 1-way ANOVA with Tukey’s post hoc test. Labeled *P* values in **G** and **I** represent the differences between the corresponding group and the control group.

To identify the upstream regulator of IGFBP7 expression and secretion, we first compared the transcriptional profiles between *IGFBP7*^hi^ and *IGFBP7*^lo^ skin ECs in psoriasis. GSEA revealed that the IFN-γ–, TNF-, and VEGF-signaling pathways were highly active in psoriatic *IGFBP7*^hi^ ECs compared with psoriatic *IGFBP7*^lo^ ECs (Figure 8A). We also treated HMEC-1 cells with psoriasis-related cytokines such as IL-17A, IL-22, IL-25, IFN-γ, TNF-α, and, VEGF-A, and among them, IFN-γ significantly upregulated the expression and secretion of IGFBP7 in HMEC-1 cells (Figure 8, B–D, and Supplemental Figure 9). Notably, conditioned medium collected from IFN-γ–treated HMEC-1 cells (IFN-γ CM) also degraded endothelial glycocalyx components, as indicated by the low MFI of HA and HS (Figure 8, E–I), and increased human CD4^+^ T cell adhesion on HMEC-1 cells in the coculture system (Figure 8, J–L). These effects were obviously reversed by pretreatment with anti-IGFBP7 antibody (Figure 8, E–L) and knockdown of IGFBP7 via siRNA transfection (Supplemental Figure 10).

As JAK/STAT signaling is the canonical pathway involved in IFN-γ signaling (33), we then evaluated its activation in IFN-γ–stimulated ECs. Western blotting showed that JAK1/2 and STAT1/3 were significantly phosphorylated by IFN-γ in HMEC-1 cells (Supplemental Figure 11, A and B). Importantly, baricitinib (a selective JAK1 and JAK2 inhibitor) and upadacitinib (a selective JAK1 inhibitor) inhibited both the mRNA and protein levels of IGFBP7 in IFN-γ–stimulated HMEC-1 cells (Supplemental Figure 11, C–G). They also blocked human CD4^+^ T cell adhesion to HMEC-1 cells that was enhanced by IFN-γ CM (Supplemental Figure 11, H and I). Taken together, our data illustrate that IFN-γ increases the expression and secretion of IGFBP7 via the JAK1/2-STAT1/3 signaling pathway in HMEC-1 cells and further promotes CD4^+^ T cell adhesion.

### Anti-IGFBP7 treatment restores the endothelial barrier and alleviates cutaneous inflammation.

**Figure 9:**
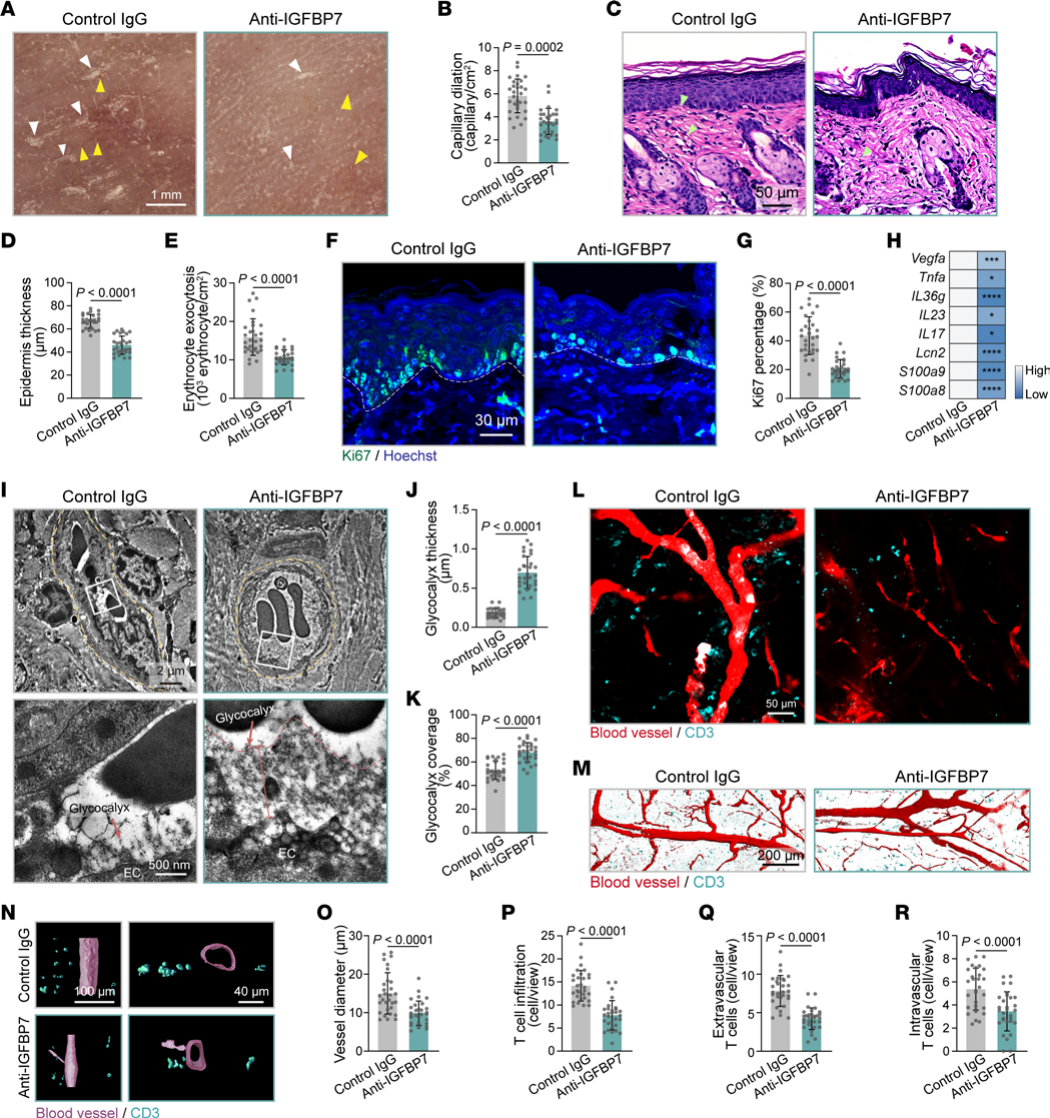
Anti-IGFBP7 treatment restores the endothelial glycocalyx and alleviates skin inflammation in IMQ mice. (**A**) Dermatoscopy of dorsal skin appearances in IMQ mice (*n* = 6 mice/group) with or without anti-IGFBP7 treatment. Yellow arrowheads indicate dilated skin capillaries; white arrowheads indicate psoriatic scales. (**B**) Quantification of dilated skin capillaries in **A** (*n* = 30 views of 6 mice/group). (**C**) Skin histology in IMQ mice (*n* = 6 mice/group) treated with or without anti-IGFBP7. Green arrowheads indicate erythrocyte exocytosis. (**D** and **E**) Quantification of epidermal thickness and erythrocyte exocytosis in **C** (*n* = 30 views of 6 mice/group). (**F**) Immunofluorescence for Ki67 (green) and Hoechst (blue) in dorsal skin tissue from IMQ mice (*n* = 6 mice/group) treated with or without anti-IGFBP7. The white dashed lines mark the interface between the epidermis and dermis. (**G**) Percentages of Ki67^+^ cells in the basal layer in **F** were quantified (*n* = 30 views of 6 mice/group). (**H**) mRNA levels of inflammatory genes in skin tissues (*n* = 6 mice/group). (**I**) TEM of skin endothelial glycocalyx in IMQ mice that received anti-IGFBP7 or control IgG (*n* = 6 mice/group). The vessel structure is highlighted by the yellow dotted line. (**J** and **K**) Endothelial glycocalyx thickness and coverage were quantified (*n* = 30 vessels of 6 mice/group). (**L**) Whole-mount immunofluorescence for skin blood vessels (red) and CD3 (cyan) (*n* = 6 mice/group). (**M** and **N**) 3D surface rendering of blood vessels and CD3 (cyan) based on whole-mount immunofluorescence. (**O** and **P**) Blood vessel diameter and infiltrated T cells in **L** were examined (*n* = 30 views/group). (**Q** and **R**) Quantification of extravascular and intravascular T cells in **N** (*n* = 30 views/group). Data are represented as mean ± SD. Significance was calculated using unpaired Student’s *t* test. *P < 0.05, ***P < 0.001, ****P < 0.0001).

Based on the pathogenic role of IGFBP7 in degrading the endothelial glycocalyx and aggravating skin inflammation, we further evaluated the possibility of targeting IGFBP7 to treat psoriasis. A monoclonal neutralizing antibody against IGFBP7 was administered to IMQ mice at the beginning of the IMQ application (Supplemental Figure 12A). Anti-IGFBP7 treatment significantly alleviated psoriasis-like skin inflammation, with reduced psoriatic scales, skin capillary dilation, erythrocyte exocytosis, epidermal thickening, keratinocyte proliferation, and mRNA levels of psoriatic cytokines and antimicrobial peptides (Figure 9, A–H, and Supplemental Figure 12, B–I). Importantly, endothelial glycocalyx structure was also restored in dorsal skin vessels of IMQ mice with anti-IGFBP7 treatment (Figure 9, I–K). Whole-mount immunofluorescence staining revealed that anti-IGFBP7 treatment normalized skin vessel structure and reduced T cell infiltration in IMQ mice (Figure 9, L–P, and Supplemental Figure 12, J–M). 3D reconstruction of immunofluorescence staining showed that extravascular and intravascular T cell infiltrations in IMQ mice were significantly reduced by anti-IGFBP7 treatment (Figure 9, Q and R, and Supplemental Figure 12, N and O). In addition, anti-IGFBP7 treatment, which started 3 days after IMQ modeling to mimic clinical settings, also dramatically alleviated psoriasis-like skin inflammation (Supplemental Figure 13). Together, these data demonstrate that anti-IGFBP7 treatment exerts therapeutic effects on endothelial dysfunction and psoriasis-like inflammation.

## Discussion

The endothelial barrier offers linkage and interaction between local tissue and circulating cells, therefore playing a leading role in mediating tissue homeostasis. In skin tissue, blood vessels branch into microcirculation, where ECs are more actively involved in material and cellular exchange. Recent studies using skin EC–specific knockout mice have demonstrated that EC dysfunction mediates neutrophil extravasation and subsequent activation, participating in psoriasis pathogenesis (34, 35). However, the low abundance and heterogeneity of skin ECs limit research progress in skin ECs and the pathological effects of blood vessels in skin diseases. Here, using scRNA-Seq, we provide a comprehensive profile of skin ECs from healthy individuals and psoriasis patients, highlight the transcriptome heterogeneity underlying EC function in the human cutaneous vasculature, and define the molecular and functional perturbations in psoriatic skin vessels, especially in skin capillaries. Endothelial glycocalyx destruction is one of the major features of endothelial dysfunction in psoriasis and is an important link during the process of immune cell extravasation. Interestingly, we identify a skin EC subset, IGFBP7^hi^ ECs, that is highly increased in the papillary blood vessels of psoriatic skin lesions. IGFBP7^hi^ ECs actively respond to psoriatic-related cytokine signaling and prepare the endothelium for immune-cell adhesion and transmigration by producing angiocrine factors. Moreover, our findings in a psoriasis-like mouse model support that administration of an anti-IGFBP7 antibody can be a feasible approach for normalizing skin vasculature and alleviating skin inflammation.

Skin vasculature is highly spatially and functionally heterogeneous, and its comprehensive alterations in inflammatory skin diseases remain unsolved. Bulk RNA-Seq averages out the signals from cell mixtures and conceals heterogeneities among cells. Although scRNA-Seq is increasingly being utilized, its application to skin vasculature has been limited due to the low abundance of ECs in skin. Here, we enriched skin ECs and performed scRNA-Seq on psoriasis patients and healthy controls, generated a single-cell transcriptome atlas of skin ECs, and identified 5 clusters (clusters A, C1, C2, P, and V) and 2 important states (IGFBP7^hi^ and IGFBP7^lo^ ECs). These data allowed us to explore unexpected biological characteristics in distinct EC clusters. We have shown that capillary skin ECs (clusters C1, C2, and P) are the dominant EC class in psoriasis, which was also confirmed in a recent study by He et al. (36). The density of capillary ECs was markedly increased in psoriasis, and their phenotypical features were prominently altered compared with those of their healthy counterparts. The top enriched pathways in psoriatic capillary ECs were mostly immune- and endothelial barrier–related and differed among clusters, including immune-cell chemoattraction, crawling, firm adhesion, barrier adjustment, extravasation (clusters C1 and C2), and activation (cluster P). Based on our previous study on the histological distribution of skin ECs, these capillary ECs (clusters C1, C2, and P) are mainly located in the superficial and intermediate plexuses in skin (6). More importantly, psoriatic capillary ECs also differed between cell states, that is, IGFBP7^hi^ and IGFBP7^lo^ ECs, as revealed by pseudotime analysis. Interestingly, IGFBP7^hi^ ECs were mainly located in the papillary dermis, where capillary dilation and immune infiltrates are prominently observed in psoriasis (37). Immune- and endothelial barrier–related functions were highly activated in IGFBP7^hi^ ECs. Therefore, using pseudotime analysis, we further distinguished an essential EC subtype with distinct locational and functional features in psoriasis.

Since dilated and tortuous blood vessels and perivascular immune infiltrates are classical hallmarks of psoriatic skin lesions (37), it is important to investigate the underlying mechanism and possible linkage between the skin vasculature and adaptive immune responses in psoriatic skin. Previous studies have claimed that upregulated adhesion molecules in blood vessels mediate inflammatory cell infiltration in psoriasis (38–40). To our surprise, using scRNA-Seq analysis and immunofluorescence staining, we found that ICAM1 was significantly increased in psoriatic skin ECs; however, the protein levels of E-selectin (SELE) and P-selectin (SELP) were not significantly changed (Supplemental Figure 14). Consistently, in another scRNA-Seq data set reported by Reynolds et al. (5), *SELE* and *SELP* were not differentially elevated in psoriatic skin ECs compared with their healthy counterparts (the fold changes were below 1.6, data not shown). The reason for the contradiction between our findings and previous publications could be due to different detection methods. Among the multifunctional changes in ECs, we demonstrate that endothelial glycocalyx destruction is one of the major functional alterations in psoriatic skin vessels, permitting the exposure of adhesion molecules and increasing T cell adhesion and subsequent extravasation. Interestingly, we also discovered that the MFIs of HA and HS were significantly reduced in the lesional vessels of patients with atopic dermatitis (AD), systemic lupus erythematosus (SLE), and scleroderma compared with those of healthy individuals (Supplemental Figure 15, A–C). Therefore, we speculate that endothelial glycocalyx degradation may be a commonly shared phenomenon in skin inflammation. These findings may further expand our understanding of the interactive relationship between skin vasculature and adaptive immune responses in the pathology of skin disease.

Our in vivo data have shown that systemically degrading the endothelial glycocalyx exacerbates immune infiltrates and tissue inflammation in the skin of IMQ mice. We have also examined other tissues/organs in mice. Notably, we found that systemically degrading the endothelial glycocalyx alone does not induce tissue inflammation in the heart, liver, spleen, kidney, and skin (data not shown), which is consistent with a previous study in which the endothelial glycocalyx in mice was degraded for 4 weeks without showing significant tissue inflammation or anatomic changes (41). In mice topically administered IMQ, no tissue inflammation was observed in visceral tissues, suggesting that IMQ-induced inflammation is mainly limited to the skin. These findings deepen our understanding of the pathogenic role of the endothelial glycocalyx in the complicated sequential process of immune-cell chemoattraction, rolling, adhesion, transmigration, and infiltration to the skin (42). Endothelial glycocalyx degradation, together with chemotaxis, dynamic changes in EC cellular junctions and extracellular matrix, and locomotion of immune cells, promotes this process. Our study focuses on a single variable at the early stage of immune-cell infiltration, where endothelial glycocalyx destruction increases the possibility of immune-cell adhesion by exposing adhesion molecules. The interactions among the endothelial glycocalyx and other variables, such as immune-cell locomotion and intercellular gap formation, warrant further investigation.

Our scRNA-Seq data sets and pseudotime trajectory of the transition of skin ECs in different states allow us to identify IGFBP7^hi^ ECs in psoriatic skin lesions. This EC subset responds to many cytokines, among which the expression and secretion of IGFBP7 are the highest after IFN-γ induction. We also examined the proportion of IGFBP7^hi^ ECs in the skin vasculature from patients with AD (Th2 dominant), SLE (IFN dominant), and scleroderma (IFN related). IGFBP7^hi^ ECs were increased in SLE (IFN dominant) and scleroderma (IFN related), but not in AD (Th2 dominant) (Supplemental Figure 15, D and E), suggesting that IGFBP7^hi^ ECs and IGFBP7-induced endothelial glycocalyx degradation may be more common in Th1-related skin diseases. This result also corresponds to our findings that IFN-γ plays a major role in upregulating IGFBP7 in ECs (Figure 8, B–D).

IGFBP7 is a secreted glycoprotein with a molecular weight of approximately 30 kDa, and although it is not an enzyme, it can sterically destroy the endothelial glycocalyx. Previous studies have demonstrated that IGFBP7 can bind to HS via its heparin-binding motif, the specific sequence of which is Lys-Ser-Arg-Lys-Arg-Arg-Lys-Gly-Lys (16), leading to shedding and degradation of the endothelial glycocalyx (17). In addition, IGFBP7 sterically regulates other structural components of the endothelial barrier. For example, by binding to the integrins α_v_β_3_, α_2_β_1_, and α_v_β1 on the surface of ECs, IGFBP7 induces stress fiber contraction, loosens VE-cadherins, and increases vascular permeability (22). IGFBP7 also interacts with various extracellular matrix components, such as type IV collagen, thereby regulating the basement membrane of the vascular wall and mediating cell transmigration (43). However, previous studies have suggested a protective role of IGFBP7 in psoriasis, which was based on the observations that IGFBP7 was downregulated in psoriatic epidermis and that keratinocyte proliferation was inhibited by IGFBP7 (31). Notably, epidermal cells express a relatively low level of IGFBP7, whereas ECs, along with smooth muscle cells and fibroblasts, are the major contributing cells of IGFBP7 expression in skin tissues (http://www.proteinatlas.org). In addition, the serum level of IGFBP7 is elevated in psoriasis patients compared with healthy individuals and is positively correlated with disease severity. Together with our data on anti-IGFBP7 treatment in psoriasis-like mice, these findings suggest that IGFBP7 is a promising therapeutic target for psoriasis.

Collectively, by constructing the single-cell transcriptome landscape of psoriatic and healthy skin ECs, we reveal the molecular and functional heterogeneity of the cutaneous vasculature in psoriatic lesions. Importantly, we identified a subset of skin ECs in psoriasis, namely, IGFBP7^hi^ ECs, that overexpress and secrete IGFBP7, destroying the endothelial glycocalyx and facilitating immune-cell adhesion and extravasation. Blocking IGFBP7 shows promising therapeutic effects for normalizing skin vasculature and alleviating skin inflammation in psoriasis. Our findings, focusing on the cutaneous vasculature, present a perspective on the occurrence and development of endothelial dysfunction, adaptive immune responses, and tissue inflammation in psoriasis. Further efforts are also needed to investigate the pathogenic contribution of different skin ECs and their interaction with neighboring cells in inflammatory skin diseases.

## Methods

For additional details on single-cell isolation, scRNA-Seq preprocessing, in vitro experiments, histology, immunofluorescence, ELISA, Western blotting, and quantitative real-time PCR, see the Supplemental Methods.

### Human samples.

Skin biopsies and peripheral blood samples were obtained from healthy individuals and patients with psoriasis vulgaris, AD, SLE, and scleroderma (Chinese Han population) at Xijing Hospital. All patients enrolled in our study had no other autoimmune or systemic diseases. Patients were not on any systemic treatment for a month and had not used any biologics. All skin lesions/tissues and the surrounding 5 cm^2^ area were not treated with any therapeutic measures for at least 2 weeks before biopsy. Two dermatologists independently determined that the dissected skin tissues were representative of their disease type. Ten healthy donors undergoing plastic surgery donated surgical skin discards for tissue immunofluorescence and TEM. Four patients with psoriasis vulgaris donated psoriatic skin lesions for scRNA-Seq. Skin lesions from 7 psoriasis patients were further processed for tissue immunofluorescence, and skin lesions from 3 psoriasis patients were collected for TEM. Three patients each with AD, SLE, or scleroderma donated skin lesions for tissue immunofluorescence. For peripheral blood samples, 32 psoriatic patients and 27 age- and sex-matched healthy donors were enrolled. Detailed clinical information for all donors is provided in Supplemental Table 1.

### scRNA-Seq and data analysis.

Single skin ECs from psoriasis patients were isolated and sequenced, generating a scRNA-Seq data set. To analyze the differences between psoriatic and healthy skin ECs, this psoriatic data set was further combined with the scRNA-Seq data set of normal human skin ECs (https://bigd.big.ac.cn/, PRJCA002692) that we recently reported (6). Detailed information on single-cell isolation, library preparation, sequencing, and data preprocessing is provided in the Supplemental Methods.

The scRNA-Seq data sets were processed and analyzed using R (version 3.6.3). The cell-density plot was examined using the ggpointdensity package (version 0.1.0) (44). DEGs of EC subsets from healthy and psoriatic skin were identified using the FindMarkers and FindAllMarkers function in the Seurat package, with a cutoff Benjamini-Hochberg adjusted *P* value of 0.05 and an average normalized log fold change of greater than 0.2. DEGs of IGFBP7^hi^ psoriatic ECs and IGFBP7^lo^ psoriatic ECs were identified using the DEsingle package (version 1.6.0), with a Benjamini-Hochberg cutoff adjusted *P* value of 0.05 and a normalized raw count fold change of greater than 2. Pathway analysis, including GO, Kyoto Encyclopedia of Genes and Genomes (KEGG), and GSEA, was achieved using the clusterProfiler package (version 3.14.3). Pseudotime analysis was carried out using the Monocle package (version 2.14.0). Transcription factor network analysis was performed using the SCENIC package (version 1.1.2-2) and the AUCell package (version 1.8.0). Data were visualized by the ggplot2 package (version 3.2.1) and Cytoscape software (version 3.9.0).

### Mouse model of psoriasis and treatments.

BALB/c mice, aged 6 to 8 weeks, were obtained from the Department of Laboratory Animal Medicine of the Fourth Military Medical University. All mice used in this study were housed in specific pathogen–free conditions with free access to a regular rodent chow diet and water. Mice were randomly assigned to different groups (*n* = 6 mice/group). The dorsal skin of the mice was shaved at least 2 days before the beginning of treatments. For the mouse model of psoriasis, daily topical application of 5% IMQ cream (INova Pharmaceuticals) was applied on the dorsal skin and on both sides of the ear skin for 5 to 8 consecutive days (29). The control mice were topically treated with the base of the IMQ.

For degradation of the endothelial glycocalyx, hyaluronidase (MilliporeSigma, H3506) and heparinase III (MilliporeSigma, H8891), which have very low levels of endotoxin (0.3 EU/mouse) and other potential contaminants so that they do not induce systemic inflammation, were used to degrade HA and HS, respectively, to shed the endothelial glycocalyx structure (9, 28, 45, 46). Mice received i.v. injection with 400 μg/mouse hyaluronidase and 100 mU/mouse heparinase III every other day for up to a total of 5 injections. rmIGFBP7 protein (10 μg/mouse; Sino Biological, 51125-M02H), which has very low levels of endotoxin (<1.0 EU/μg) and other potential contaminants and does not induce systemic inflammation, was i.v. injected every other day for up to 5 injections to increase the circulating level of IGFBP7. The control mice were injected with the same volume of saline. For the combination of the mouse model of psoriasis and endothelial glycocalyx degradation or an increase in IGFBP7, i.v. injection started 4 days before the topical application of IMQ. IMQ application lasted for 5 consecutive days.

For anti-IGFBP7 therapy, 2 treatments with different timings were carried out. In one treatment, the first dose of the therapy started 1 day before IMQ application (Supplemental Figure 11A). The control mice were treated with the same amount of rabbit IgG. In the other treatment, the first dose of the therapy started on day 3 of IMQ application (Supplemental Figure 12A), as previously reported (47). The control mice were treated with the same amount of rabbit IgG. Both treatments used a neutralizing IGFBP7 antibody (100 μg/mouse; ABclonal, catalog A4615), which was i.p. injected every other day for up to a total of 3 treatments, as previously reported (48).

For all murine experiments, the dorsal skin lesions were magnified and recorded via dermatoscopy (HEINE, delta 20 plus). Biological samples were harvested at the end of the treatment.

### TEM and SEM.

TEM and SEM analyses of the endothelial glycocalyx were performed as previously described, with some modifications (49–51). Briefly, freshly collected human skin tissues were sliced into small pieces; immediately immersed in a phosphate-buffered fixative (pH 7.3) composed of 2% glutaraldehyde, 0.05% Alcian blue 8GX, and 30 mmol/L MgCl_2_ for 1 hour; and then soaked in phosphate-buffered fixative with 2% lanthanum nitrate (MilliporeSigma, catalog 331937), 2% glutaraldehyde, and 2% sucrose overnight (4°C).

For SEM analysis, tissues were washed in alkaline (0.03 mol/L NaOH) 2% sucrose solution. The specimens were then dehydrated through a graded ethanol series. After ion sputtering (Hitachi E-1045), the specimens were examined using SEM (FEI Inspect).

For TEM analysis, the specimens were postfixed with 1% aqueous osmium tetroxide and 1% lanthanum nitrate (MilliporeSigma) for 1 hour, dehydrated through a graded ethanol series, and embedded in Epon812. Ultrathin sections (60–90 nm) stained with uranyl acetate (15 minutes) and lead citrate (2 minutes) were then examined using TEM (FEI Tecnai G2). Quantitative assessment of the endothelial glycocalyx thickness and the endothelial glycocalyx occupation area of the vascular lumen area was performed on 9 randomly chosen blood vessels using TEM images from skin tissues of healthy individuals (*n* = 3 skin samples/group) and psoriasis patients (*n* = 3 skin samples/group) using ImageJ software (NIH).

### Flow assay.

Peripheral blood freshly collected from healthy donors was lysed in NH_4_Cl buffer to discard red blood cells and then incubated with anti-CD4 antibody–coated micromagnetic beads (Miltenyi Biotech, catalog 130-045-101) for 15 minutes at 4°C. After incubation, the sample was added to a sterile tube containing 2 mL of PBS/EDTA buffer and then centrifuged for 10 minutes at 300*g*. Then the cells were resuspended in 500 μL of PBS/EDTA buffer and added to a magnetic separation column in the magnetic field of a mini magnetic-activated cell-sorting separator (Miltenyi Biotech). After positive selection, the column was washed 3 times with PBS/EDTA buffer for cell collection. The purity of these selected cells was more than 90%, as confirmed by flow cytometry using a BD LSR Fortessa system (BD). CD4^+^ T cells were stained with 1 μM calcein-AM (Invitrogen, catalog C1429) for 15 minutes at 37°C for further experiments.

HMEC-1 cells were seeded on the bottom of a single-channel flow chamber (ibidi, μ-Slide I^0.6^ Luer) and cultured under static conditions until the cells reached 100% confluence. HMEC-1 cells were treated with 100 μg/mL hyaluronidase (MilliporeSigma) and 50 mU/mL heparinase III (MilliporeSigma) or 400 μg/mL rhIGFBP7 protein (Sino Biological, catalog 13100-H08H) for 3 hours. Then the medium was replaced with fresh cell-culture medium according to the manufacturer’s instructions. The CD4^+^ T cell suspension (1 × 10^4^ cells/mL) was flushed through the flow chamber using a high-precision peristaltic pump (Longer Precision Pump Co., BT100-2J) at a controlled flow rate of 1 mL/min, based on the manufacturer’s instructions. The flow of CD4^+^ T cells was filmed under an immunofluorescence microscope (Olympus, IX71). The adhered CD4^+^ T cells were counted at the end of the flow assay.

### Whole-mount immunofluorescence staining.

For visualization of blood vessels and CD3^+^ T cells in mouse ear skin, whole-mount immunofluorescence staining was carried out as previously described with minor modifications (52). In brief, mice were i.v. injected with 100 μL of TRITC-dextran (500 kDa, 10 mg/mL; MilliporeSigma, catalog 52194) 10 minutes before sacrifice. The dorsal side of the ear was carefully dissected, fixed in 4% paraformaldehyde, permeabilized in 0.3% Triton X-100 in PBS (PBST), and blocked with 3% skim milk in PBST. The ear skin was incubated with an anti-mouse CD3 antibody (Abcam, catalog ab135372, 1:200) at 4°C for 24 hours. After being washed in 0.05% Tween-20 in PBS, the samples were incubated with secondary antibodies at room temperature for 2 hours before washing and mounting. Images were captured by a confocal microscope (Carl Zeiss, LSM880). Negative controls that were stained with secondary antibodies were included in all experiments. Image analysis, including the calculation of blood vessel diameter and infiltrated T cells, was performed using ZEN software (Carl Zeiss, version 1.1.2.0) and ImageJ software. 3D reconstruction was performed using Imaris software (version 7.4.2).

### Data and code availability.

The scRNA-Seq data sets were deposited at the National Genomics Data Center (PRJCA007038; PRJCA002692). This study did not generate new unique code.

### Statistics.

Each experiment was performed at least 3 times. All data are presented as the mean ± SD. Statistical analyses of the data were performed using GraphPad Prism (version 9.0.2) and R. The differences between the 2 groups were compared by unpaired, 2-tailed Student’s *t* test. For comparisons of more than 2 groups, 1-way ANOVA was performed. One-way ANOVA followed by Tukey’s post hoc test was used to compare a group with every other group, and 1-way ANOVA followed by Dunnett’s test was used to compare each group with the control group. Correlation analysis was performed using Spearman’s rank correlation test. *P* < 0.05 was considered statistically significant.

### Study approval.

For human studies, written, informed consent from the donors was obtained. Human samples were used in full agreement with the approval of the Ethics Committee of Xijing Hospital at the Fourth Military Medical University (KY20183019-1). Animal procedures were performed according to NIH guidelines and were approved by the Review Committee for the Use of Animals of the Fourth Military Medical University.

## Author contributions

QL, GW, and ED conceived the project and designed the experiments. QL performed all experiments and data analyses unless otherwise noted. SS provided technical assistance and edited the manuscript. ZZ, JC, JH, and YB provided human samples and technical assistance and helped perform scRNA-Seq. JH, BL, ED, and GW obtained the funding for this study. QL wrote the manuscript with input and revision from GW, ED, and SS. All authors read and approved the final manuscript. All of the authors approved the final version of the manuscript.

## Supplementary Material

Supplemental data

Supplemental data set 1

Supplemental data set 2

Supplemental video 1

Supplemental video 2

Supplemental video 3

## Version 1 03/14/2023

In-Press Preview

## Version 2 05/01/2023

Electronic publication
