# Supplementary material for: An IGFBP7hi endothelial cell subset drives T cell extravasation in psoriasis via endothelial glycocalyx degradation
Source: J Clin Invest. 2023 May 1;133(9):e160451. doi: 10.1172/JCI160451 (PMC10145935; doi:10.1172/JCI160451)
Supplement: Supplemental data [file jci-133-160451-s077.pdf]

Supplemental Materials

This PDF file includes:

Supplemental Methods

References

Supplemental Figures and Legends 1 to 15

Supplemental Tables 1 to 3

Captions for Supplemental Movies 1 to 3

Captions for Supplemental Data 1 to 2

Other Supplemental Materials for this manuscript include the following:

Supplemental Movies 1 to 3

Supplemental Data 1 to 2 (Excel format)

Supplemental Methods

1. Single-cell isolation, sequencing, and data preprocessing

Single skin endothelial cells from psoriasis patients were isolated and sequenced, generating a single-cell RNA sequencing (scRNA-seq) dataset. To analyze the differences between psoriatic and healthy skin vascular endothelial cells (ECs), this psoriatic dataset was further combined with the scRNA-seq dataset of normal human skin ECs (National Genomics Data Center, https://ngdc.cncb.ac.cn, PRJCA002692) that we recently reported (1). Detailed information on (i) single endothelial cell isolation from psoriasis patients, (ii) library preparation and sequencing of the psoriatic scRNA-seq dataset, and (iii) quality control, integration, and batch correction of healthy and psoriatic scRNA-seq datasets were as follows.

(i) Single endothelial cell isolation from psoriasis patients. Endothelial cells, collected from the skin biopsies of four psoriasis patients (Supplemental Table 1), were isolated by a magnetic- and fluorescence-activated cell sorting strategy as previously described (1). In brief, human skin tissues were cut into small pieces immediately after surgery and were transferred into RPMI-1640 medium (Gibco, cat. No. 21870092) containing 2.5 mg/mL Dispase II (Gibco, cat. No. 17105041) overnight (4 °C). After removing the epidermis layer, the dermis tissue was homogenized and digested in RPMI-1640 medium containing 1 mg/mL type IV collagenase (Worthington, cat. No. LS004188), 0.2 mg/mL hyaluronidase (Sigma, cat. No. H3506), 0.2 mg/mL Dnase I (Sigma, cat. No. DN25), and 10 mM HEPES (Gibco, cat. No. 15630080) for 40 min (38 °C, 265 r/min). 10 mL of precooled washing buffer (4 °C) was added to the cell suspension to terminate the digestion. Then, the cell suspension was first used to enrich $CD31^+$ cells via the CD31 MicroBead Kit (Miltenyi Biotec, cat. No. 130-091-935) according to the manufacturer's instructions. The $CD31^+$ cells obtained by magnetic-activated cell sorting were further processed to select live $CD31^+$ and $CD45^-$ endothelial cells via fluorescence-activated cell sorting using a MoFlo XDP cell sorter (Beckman Coulter). Cells were incubated with anti-human CD31 antibody conjugated with FITC (BioLegend, cat. No. 303103), anti-human CD45 antibody conjugated with APC (BioLegend, cat. No. 304011), and 7-AAD (BioLegend, cat. No. 420403). Flow cytometric data were analyzed using FlowJo software. Cell viability was examined by Trypan blue staining (Sigma, cat. No. T8154) and controlled with a threshold set at > 90%.

(ii) Library preparation and sequencing of the psoriatic scRNA-seq dataset. Freshly sorted skin endothelial cells from psoriasis patients were immediately processed for library preparation and sequencing by Genergy Biotechnology (Shanghai) Co., Ltd., according to the 10X Genomics guidelines. The sample was sequenced with a cell number of 3,999, a sequencing depth of 89,065 reads per cell, a sequencing saturation rate of 80.5%, and a median of 2,481 genes per cell. Demultiplexing, read alignment, and quality control were performed at Genergy Biotechnology (Shanghai) Co., Ltd., using the CellRanger package. The gene expression matrix was further imported into R (version 3.6.3) for quality control, data integration, batch correction, and further analysis.

(iii) Quality control, integration, and batch correction. For quality control, genes expressed in fewer than 3 cells and cells expressing fewer than 200 genes were removed from the gene expression matrix. Then, low-quality cells were filtered out using the quickPerCellQC function (nmads = 1.5) in the Scater package (version 1.14.6). To integrate the scRNA-seq datasets

of healthy and psoriatic skin ECs, 2,000 endothelial cells from the healthy scRNA-seq dataset (1) were randomly subsampled, and the FindIntegrationAnchors and IntegrateData functions in the Seurat package (version 3.1.2) were applied. For batch effect correction, fast MNN in the SeuratWrappers package (version 0.1.0) was performed.

To annotate cell clusters in the psoriatic scRNA-seq dataset, FindTransferAnchors and TransferData functions in the Seurat package were used, which is a well-accepted data integration method (2). The cell annotations in the scRNA-seq dataset of healthy ECs were used as reference data. Psoriatic single cells with prediction scores below 0.5 were excluded (8.65% of cells). We further examined possible contamination of other cell types. One cell with no expression of endothelial markers (*PECAM1* and *CDH5*) and positive expression of smooth muscle cells (*RGS5*) was filtered out. Other non-endothelial cells, defined by positive expression as fibroblasts (*COL1A1*), pericytes (*PDGFRA* and *PDGFRB*), or red blood cells (*HBA1*, *HBA2*, and *HBB*), and no expression of *PECAM1* and *CDH5*, were not found.

For further analysis, scRNA-seq data were normalized and scaled. The variabilities in the number of counts, features, and percentages of mitochondrial and ribosomal genes were regressed out. The detailed methods of further analysis are provided in the Methods section of the main text.

2. Histology

The dorsal skin tissues of the mice were fixed in formalin, embedded in paraffin, and sectioned at 4 μm. Sections of skin tissues were deparaffinized, stained with H&E solution, and mounted with resinous mounting medium. H&E staining images were acquired with a slide scanner (Olympus Corporation, VS120), and the average epidermal thickness was measured by ImageJ software (version 2.0.0).

3. EC culture and treatments

The human dermal microvascular endothelial cell line HMEC-1 was purchased from ATCC (CRL-3243) and cultured in MCDB131 medium (Gibco, cat. no. 10372019) with 10 ng/mL EGF (Sino Biological, cat. no. GMP-10605-HNAE), 1 μg/mL hydrocortisone (Solarbio, cat. no. IH0100), 10 mM glutamine, and 10% fetal bovine serum (Biological Industries, cat. no. 04-001-1ACS) at 37 °C in 5% CO_2.

First, to evaluate the function of IGFBP7 in ECs, HMEC-1 cells were divided into three groups. HMEC-1 cells were treated with 400 ng/mL recombinant human IGFBP7 protein (rhIGFBP7; Sino Biological, cat. no. 13100-H08H) for 3 h. HMEC-1 cells treated with 100 μg/mL hyaluronidase (Sigma, cat. no. H3506) and 50 mU/mL heparinase III (Sigma, cat. no. H8891) were endothelial glycocalyx-degraded and used as the positive control. HMEC-1 cells treated with the same volume of PBS were used as the negative control. These HMEC-1 cells were further processed for examination of hyaluronic acid (HA) and heparan sulfate (HS) via immunofluorescence staining and cell adhesion assays. The expression levels of *SELE*, *SELP*, and *ICAM1* were detected by quantitative real-time PCR (qPCR) and western blotting.

Second, to identify the upstream cytokines leading to upregulated IGFBP7 expression, recombinant human IL-17A (Sino Biological, cat. no. CT047-HNAE), IL-22 (Sino Biological, cat. no. 13059-HNAE), IL-25 (Sino Biological, cat. no. 10096-H01H), IFN-γ (Sino Biological, cat. no.

11725-HNAS), TNF-α (Sino Biological, cat. no. 10602-HNAE), and VEGF-A (Sino Biological, cat. no. 29714-HNAH) at a series of concentrations (20, 50, and 100 ng/mL) were added to the culture medium of HMEC-1 cells. IGFBP7 expression was detected via qPCR and immunofluorescence staining. The secretion of IGFBP7 was examined via ELISA.

Third, to test the critical role of IGFBP7 in endothelial glycocalyx degradation and cell adhesion, IGFBP7 was blocked or knocked down in the presence of IFN-γ. In one experiment, a neutralizing antibody was used based on a reported study (3). The conditioned medium of HMEC-1 cells pretreated with 100 ng/mL IFN-γ was collected after 48 h (this conditioned medium was further referred to as IFN-γ-treated HMEC-1 cell-conditioned medium). The conditioned medium of HMEC-1 cells pretreated with PBS was obtained (this conditioned medium was further referred to as PBS-treated HMEC-1 cell-conditioned medium). Fresh HMEC-1 cells were divided into three groups. The three groups of HMEC-1 cells were stimulated with (i) PBS-treated HMEC-1 cell-conditioned medium and 25 μg/mL control IgG, (ii) IFN-γ-treated HMEC-1 cell-conditioned medium and 25 μg/mL control IgG, or (iii) IFN-γ-treated HMEC-1 cell-conditioned medium and 25 μg/mL IGFBP7-neutralizing antibody (Sino Biological, cat. no. 13100-MM01) for 3 h. In the other experiment, si-IGFBP7 (forward, 5'-ccaaggacaucuggaaugutt-3'; reverse, 5'-acauuccagauguccuuggtt-3') was transfected into HMEC-1 cells via Lipofectamine™ 3000 (Invitrogen, cat. no. L3000075) according to the manufacturer's protocol (4). The efficiency of IGFBP7 knockdown was confirmed by qPCR and western blotting. The conditioned medium of HMEC-1 cells transfected with si-IGFBP7 and treated with 100 ng/mL IFN-γ was collected (this conditioned medium was further referred to as si-IGFBP7+IFN-γ conditioned medium). The conditioned media of HMEC-1 cells transfected with si-NC and treated with 100 ng/mL IFN-γ (further referred to as si-NC+IFN-γ conditioned medium) or transfected with si-NC and treated with PBS (further referred to as si-NC+ PBS conditioned medium) were collected. Fresh HMEC-1 cells were divided into three groups. The three groups of HMEC-1 cells were stimulated with (i) si-NC+PBS conditioned medium, (ii) si-NC+IFN-γ conditioned medium, and (iii) si-IGFBP7+IFN-γ conditioned medium for 3 h. The HMEC-1 cells in the two experiments were further processed for examination of HA and HS via immunofluorescence staining and cell adhesion assays.

Finally, to investigate the upstream mechanism by which IFN-γ modulates IGFBP7 expression, two JAK inhibitors, baricitinib and upadacitinib, were used. The efficiency of baricitinib and upadacitinib in inhibiting the JAK-STAT signaling pathway was confirmed via western blotting. HMEC-1 cells were divided into four groups: (i) PBS and DMSO treatment, (ii) 100 ng/mL IFN-γ and DMSO, (iii) 100 ng/mL IFN-γ and 10 μM baricitinib (MedChemExpress, cat. no. HY-15315), and (iv) 100 ng/mL IFN-γ and 10 μM upadacitinib (MedChemExpress, cat. no. HY-19569). HMEC-1 cells were collected to examine the expression of IGFBP7 via qPCR, immunofluorescence staining, and western blotting. In addition, to evaluate the role of the JAK-STAT signaling pathway in mediating cell adhesion, the conditioned media of the four groups of HMEC-1 cells mentioned above were obtained and further added to fresh HMEC-1 cells for 3 h. HMEC-1 cells were then processed for cell adhesion assays.

4. Cell adhesion assays

Coculture and flow assays were carried out to examine the ability of HMEC-1 cells to adhere to $CD4^+$ T cells.

Peripheral blood freshly collected from healthy donors was lysed in NH_4Cl buffer to discard red blood cells and then was incubated with anti-CD4 antibody-coated micromagnetic beads (Miltenyi Biotech, cat. no. 130-045-101) for 15 min at 4 °C. After incubation, the sample was added at 2 mL per sterile tube using PBS/EDTA buffer and then centrifuged for 10 min at 300×g. Next, the cells were resuspended in 500 µL of PBS/EDTA buffer and added to a magnetic separation column in the magnetic field of a mini magnetic-activated cell sorting separator (Miltenyi Biotech). After positive selection, the column was washed three times with PBS/EDTA buffer for cell collection. The purity of these selected cells was more than 90%, as confirmed by flow cytometry using a BD LSR Fortessa system (Becton, Dickinson and Company). $CD4^+$ T cells were stained with 1 µM calcein-AM (Invitrogen, cat. no. C1429) for 15 min at 37 °C for further experiments.

For the static coculture assay, HMEC-1 cells were cultured in 24-well plates. After different treatments (as mentioned above), HMEC-1 cells were washed with PBS. $CD4^+$ T cells were added to wells (1×10^5 cells per well), together with a new culture medium (MCDB131 and RPMI-1640, Gibco). After 5 h of coculture, each well was gently washed with PBS 5 times to remove $CD4^+$ T cells that did not adhere to HMEC-1 cells. Five different fields in each well were examined under an immunofluorescence microscope (Nikon Eclipse Ti-S). Cell counts of the images were analyzed by ImageJ software.

For the flow assay, HMEC-1 cells were seeded on the bottom of a single channel flow chamber (ibidi, µ-Slide $I^{0.6}$ Luer) and cultured under static conditions until the cells reached 100% confluence. HMEC-1 cells were treated with 100 µg/mL hyaluronidase (Sigma) and 50 mU/mL heparinase III (Sigma) or 400 µg/mL rhIGFBP7 protein (Sino Biological, cat. no. 13100-H08H) for 3 h. Then, the medium was replaced with fresh cell culture medium according to the manufacturer's instructions. The $CD4^+$ T cell suspension (1×10^4 cells/mL) was flushed through the flow chamber using a high-precision peristaltic pump (Longer Precision Pump Co., Ltd., BT100-2J) at a controlled flow rate of 1 mL/min, based on the manufacturer's description. The flow of $CD4^+$ T cells was filmed under an immunofluorescence microscope (Olympus, IX71). The adhered $CD4^+$ T cells were counted at the end of the flow assay.

5. Immunofluorescence staining

Skin lesions from patients with psoriasis vulgaris, atopic dermatitis (AD), systemic lupus erythematosus (SLE), and scleroderma and skin tissues from healthy donors (Supplemental Table 1) and mice were obtained; fixed in 4% paraformaldehyde; and embedded in paraffin. For immunofluorescence staining, tissue sections were deparaffinized and subjected to antigen retrieval with Tris-EDTA buffer (pH 9.0). After blocking non-specific staining with 5% goat serum in PBS for 1 h at room temperature, the sections were incubated with primary antibodies (Supplemental Table 2) overnight (4 °C) and then were incubated with secondary antibodies for 1 h (room temperature). Hoechst staining (1:1000) was used to indicate the cell nucleus (10 min, room temperature).

HMEC-1 cells seeded in confocal dishes were fixed in 4% paraformaldehyde for 15 min, blocked with 5% goat serum in PBS for 1 h at room temperature, and incubated with primary antibodies (Supplemental Table 2) overnight (4 °C). Dishes were washed gently with PBS before incubation with secondary antibodies and Hoechst staining. Dishes were preserved in PBS at 4 °C

before imaging.

For visualization of blood vessels and $CD3^+$ T cells in murine ear skin, whole-mount immunofluorescence staining was carried out as previously described with minor modifications (5). In brief, mice were intravenously injected with 100 μL of TRITC-dextran (500 kDa, 10 mg/mL; Sigma, cat. no. 52194) 10 min before sacrifice. The dorsal side of the ear was carefully dissected, fixed in 4% paraformaldehyde, permeabilized in 0.3% Triton X-100 in PBS (PBST), and blocked with 3% skim milk in PBST. The ear skin was incubated with an anti-mouse CD3 antibody (Abcam, cat. no. ab135372, 1:200) at 4 °C for 24 h. After being washed in 0.05% Tween-20 in PBS, the samples were incubated with secondary antibodies at room temperature for 2 h before washing and mounting.

Images of all the specimens were captured by a confocal microscope (Carl Zeiss, LSM880). Negative controls that were stained with secondary antibodies were included in all experiments. Image analysis, including quantifications of MFI, percentage of $Ki67^+$ cells in the basal layer of the epidermis, blood vessel diameter, and infiltrated T cells, were performed by ZEN software (Carl Zeiss, version 1.1.2.0) and ImageJ software. 3D reconstruction, MFI quantification, and volume calculation were performed using Imaris software (version 7.4.2).

6. ELISA

Serum levels of HA, HS, and IGFBP7 in peripheral blood samples from psoriasis patients and healthy individuals or conditioned medium of HMEC-1 cells were quantified using a human hyaluronan Quantikine ELISA kit (R&D Systems, cat. no. DHYALO), human HS ELISA kit (Elabscience, cat. no. E-EL-H2364), and human IGFBP-7 ELISA kit (Elabscience, cat. no. E-EL-H0447). The concentration of IGFBP7 in mouse serum was measured using a mouse IGFBP-rp1/IGFBP-7 ELISA kit (RayBio, cat. no. ELM-IGFBPRP1). All ELISAs were performed according to the manufacturers' protocol.

7. Western blotting

Western blotting was performed using standard methods. In brief, cells were washed in PBS and lysed in RIPA lysis buffer (Beyotime) with a protease inhibitor cocktail (Roche) on ice for 20 min. The total protein concentration was quantified with a BCA Protein Assay kit (Tiangen, cat. no. PA115). Equal amounts of protein were separated on SDS–PAGE gels and transferred to PVDF membranes (Millipore). The membrane was blocked with 5% skim milk, incubated with primary antibodies (Supplemental Table 2) overnight at 4 °C, and then incubated with HRP-conjugated secondary antibodies for 1 h at room temperature. Blots were visualized by the ChemiDocTM MP Imaging System (Bio-Rad) and analyzed via Image Lab software (Bio-Rad, version 6.0).

8. qPCR

Total RNA from skin specimens or cultured cells was extracted by TRIzol reagent (Invitrogen) and then reverse-transcribed to cDNA using the PrimeScript RT Reagent Kit (TaKaRa, cat. no. RR047A). qPCR was performed using SYBR Premix Ex Taq II (TaKaRa, cat. no. RR820A) on an iQ5 PCR Detection System (Bio-Rad). The relative mRNA expression levels were calculated based on the $2^{-\Delta\Delta CT}$ method. The sequences of the primers used in this study are provided in Supplementary Table 3.

References

1. Li Q, et al. Single-cell transcriptome profiling reveals vascular endothelial cell heterogeneity in human skin. *Theranostics*. 2021;11(13):6461–6476.
2. Stuart T, et al. Comprehensive integration of single-cell data. *Cell*. 2019;177(7):1888-1902.e21.
3. Lee WC, et al. Plasmodium-infected erythrocytes induce secretion of IGFBP7 to form type II rosettes and escape phagocytosis. *Elife*. 2020;9:e51546.
4. Zhang J, et al. Decreasing growth differentiation factor 15 promotes inflammatory signals and neutrophil infiltration in psoriasis models. *J Invest Dermatol*. 2022;S0022-202X(22)01885–1.
5. Zhang Y, et al. Heterogeneity in VEGFR3 levels drives lymphatic vessel hyperplasia through cell-autonomous and non-cell-autonomous mechanisms. *Nat Commun*. 2018;9(1):1296.

Supplemental Figures and Figure Legends

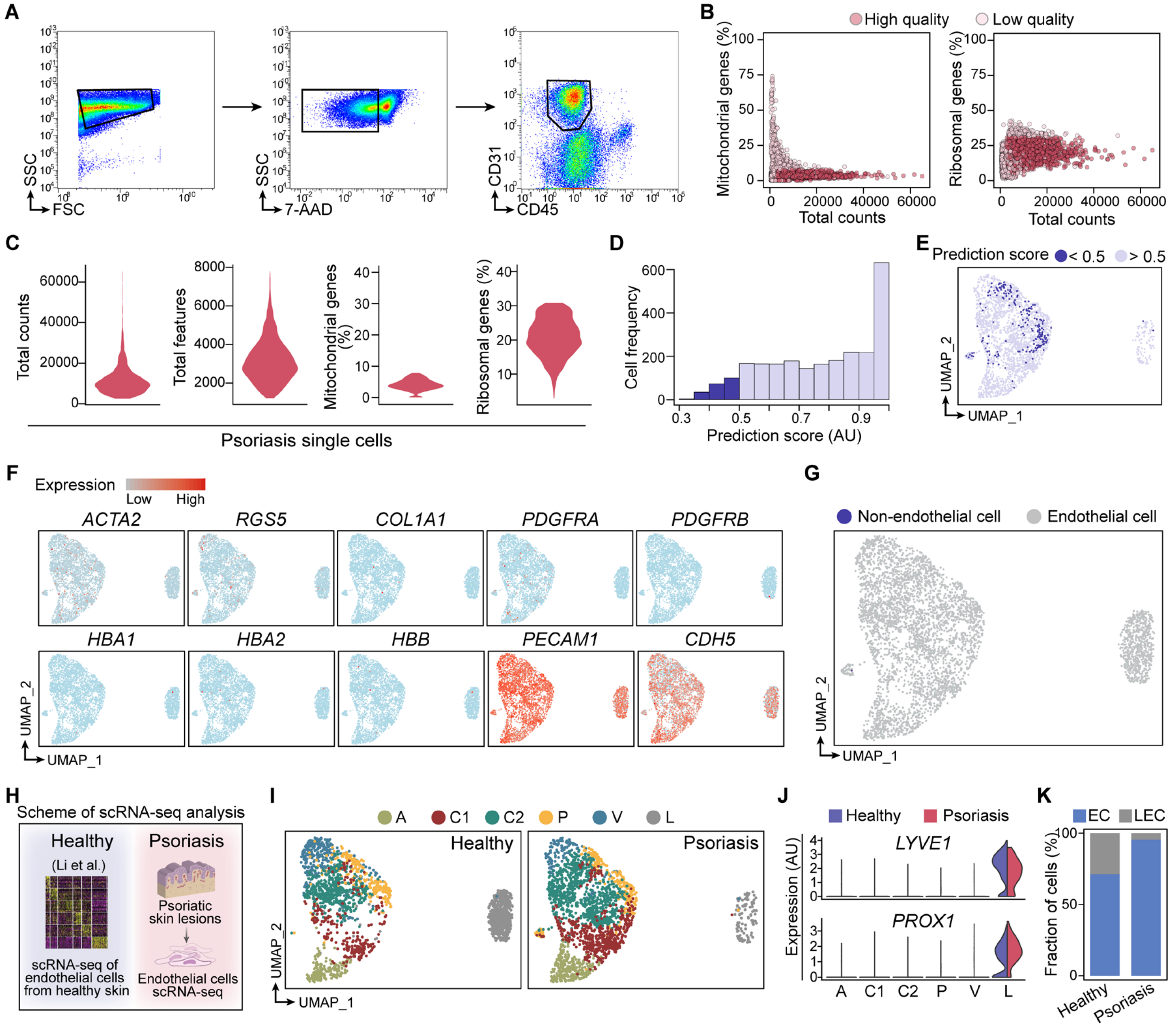

Supplemental Figure 1. Sorting of psoriatic skin ECs and processing of scRNA-seq dataset.

(**A**) Gating logic to sort live $CD31^{+}$ $CD45^{-}$ cells (endothelial cells) from psoriatic skin tissues via flow cytometry. (**B**) Distribution of total counts, percentage of mitochondrial genes, and percentage of ribosomal genes in psoriasis single cells before quality control. Each circle represents a cell. High-quality cells are colored dark red, and low-quality cells are colored light red. (**C**) The total counts, total features, mitochondrial gene percentage, and ribosomal gene percentage in the psoriasis scRNA-seq dataset after quality control. (**D**) The frequency of prediction scores in psoriatic single cells. Healthy skin ECs (Li et al.) were used as a reference dataset. (**E**) Distribution of psoriatic cells with prediction scores below and above 0.5. (**F**) Expression of markers of non-endothelial cells, including smooth muscle cells (*ACTA2* and *RGS5*), fibroblasts (*COL1A1*), pericytes (*PDGFRA* and *PDGFRB*), red blood cells (*HBA1*, *HBA2*, and *HBB*), and ECs (*PECAM1* and *CDH5*), in the psoriasis scRNA-seq dataset. (**G**) UMAP plot showing ECs and non-endothelial cells. (**H**) Schematic of scRNA-seq analysis of endothelial cells from healthy and psoriatic skin, the former of which was extracted from our previously reported

study (Li et al.). (**I**) UMAP plots showing the clusters of healthy and psoriatic skin endothelial cells, including ECs (clusters A, C1, C2, P, and V) and lymphatic endothelial cells (cluster L). Dots in different colors represent cells in corresponding clusters, which are cluster A (arteriole ECs); clusters C1, C2, and P (capillary ECs); cluster V (venule ECs); and cluster L (lymphatic endothelial cells). (**J**) Violin plots showing the expression of lymphatic endothelial cell markers (*PROX1* and *LYVE1*) in healthy and psoriatic endothelial cells. (**K**) Bar plot showing the proportion of ECs and lymphatic endothelial cells (LECs) in healthy and psoriatic skin.

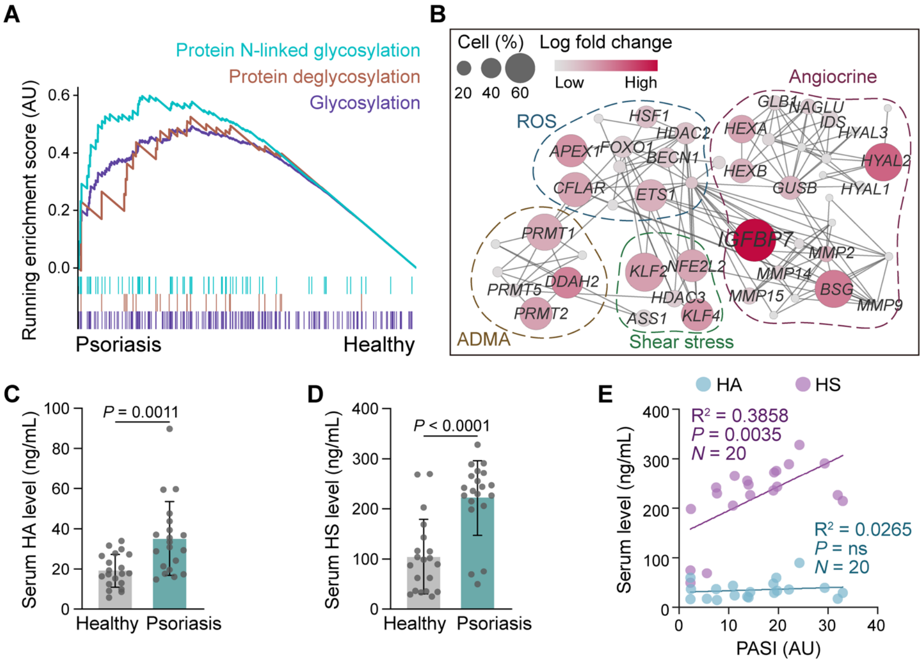

Supplemental Figure 2. Degradation of the endothelial glycocalyx of skin blood vessels in psoriasis patients.

(**A**) GSEA enrichment plot showing the pathways enriched in psoriatic capillary ECs compared to healthy capillary ECs. (**B**) Dot plot showing the expression of genes related to endothelial glycocalyx degradation in psoriatic capillary ECs. Color indicates the log fold change between the average expression of psoriatic capillary ECs vs. healthy capillary ECs. Size indicates the percentage of cells in which the gene is expressed. (**C** and **D**) Serum concentrations of HA and HS in peripheral blood from healthy and psoriatic donors (n = 20 blood samples/group). (**E**) Linear regression analysis of the correlation between the serum HA or HS level and disease severity (psoriasis area and severity index (PASI) score) in psoriasis patients. Data in **C** and **D** represent the mean ± SD and were analyzed using unpaired Student's t test.

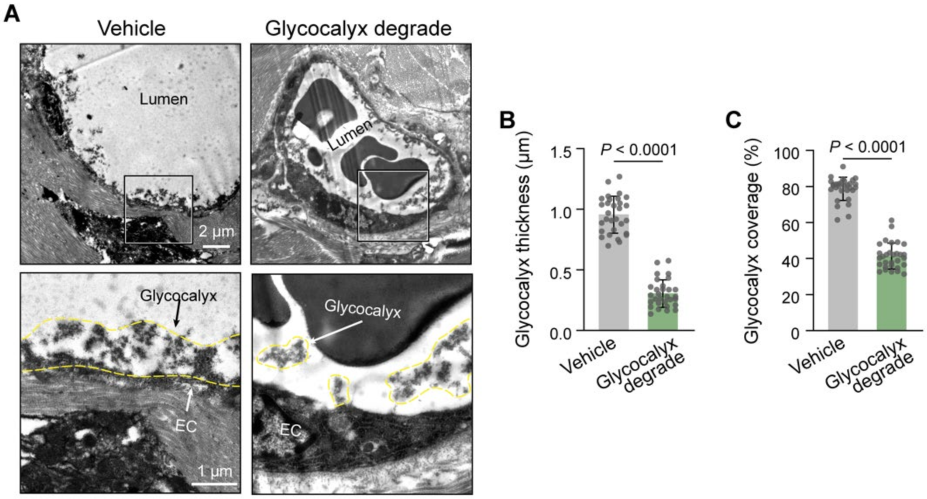

Supplemental Figure 3. Degradation of the endothelial glycocalyx of skin blood vessels in mice.

(**A**) Transmission electron micrographs showing that the endothelial glycocalyx structure in skin blood vessels from control mice (*n* = 6 mice/group) was successfully degraded by the injection of hyaluronidase and heparinase III. The endothelial glycocalyx is highlighted by the yellow dotted line. (**B** and **C**) Quantification of endothelial glycocalyx thickness and coverage (*n* = 30 vessels of 6 mice/group) in **A**. All data represent the mean ± SD. Analysis was performed using the unpaired Student's t test.

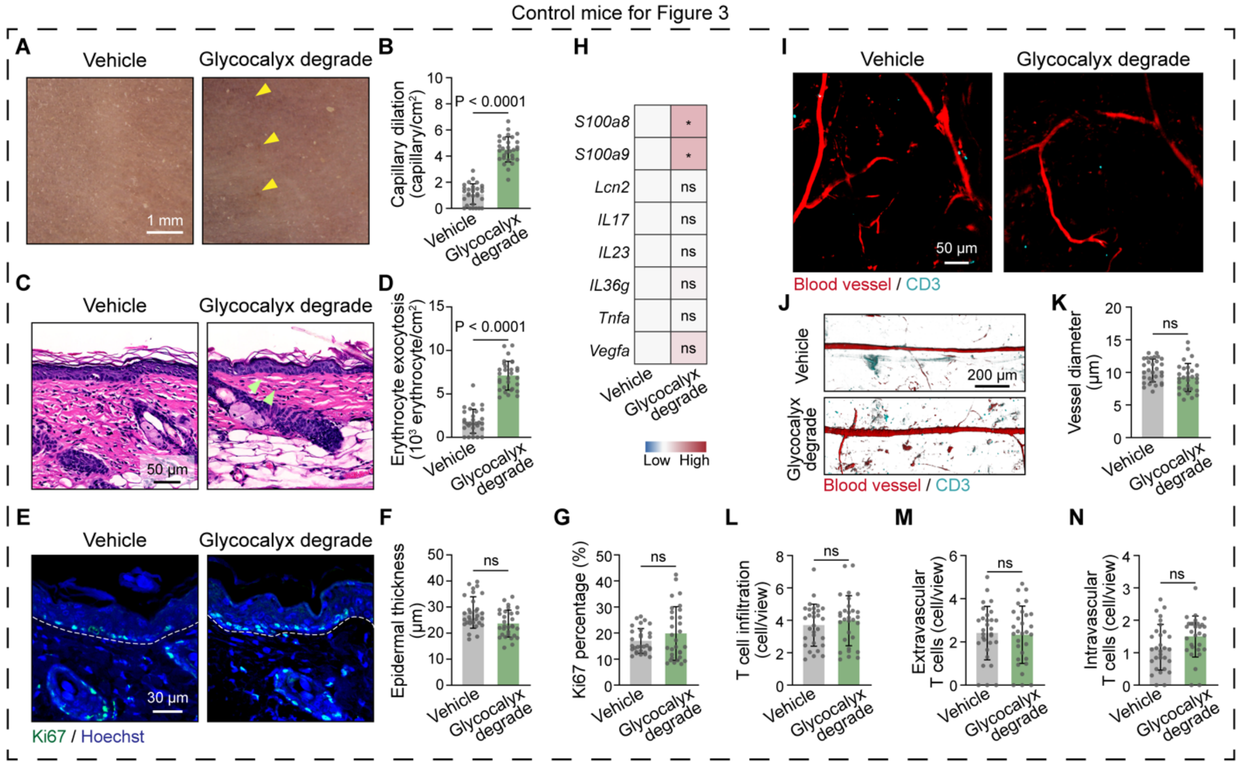

Supplemental Figure 4. Control mice with or without degradation of the endothelial glycocalyx.

(**A**) Dermatoscopy shows the dorsal skin appearance of control mice (n = 6 mice/group) with or without endothelial glycocalyx degradation. Yellow arrowheads, which point at red dots, indicate dilated skin capillaries. (**B**) Quantification of dilated skin capillaries in **A**. (**C**) H&E staining of dorsal skin tissue from control mice (n = 6 mice/group) with or without endothelial glycocalyx degradation. Green arrowheads indicate erythrocyte exocytosis. (**D**) Quantification of erythrocyte exocytosis in **C**. (**E**) Immunofluorescence for Ki67 (green) and Hoechst (blue) of dorsal skin tissue from control mice (n = 6 mice/group) with or without endothelial glycocalyx degradation. The dashed white line marks the interface between the epidermis and dermis. (**F** and **G**) Quantification of epidermal thickness and the percentage of $Ki67^{+}$ cells in the basal layer in **E**. (**H**) Heatmap showing the transcriptional levels of inflammatory genes in the dorsal skin of control mice with or without endothelial glycocalyx degradation via qPCR. (**I**) Whole-mount immunofluorescence staining for blood vessels (red) and CD3 (cyan) in ear skin from control mice (n = 6 mice/group) with or without endothelial glycocalyx degradation. (**J**) 3D surface rendering of blood vessels (red) and CD3 (cyan) based on whole-mount immunofluorescence staining. (**K**–**N**) The average blood vessel diameter, infiltrated T cells, extravascular T cells, and intravascular T cells were examined in **I** (n = 30 views of 6 mice/group). All data represent the mean ± SD. Analysis was performed using the unpaired Student's t test (* P<0.05).

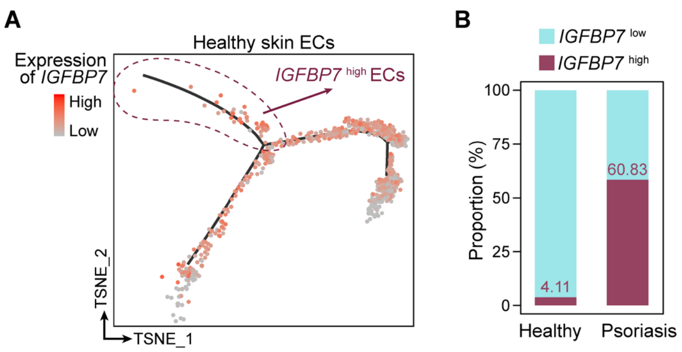

Supplemental Figure 5. *IGFBP7*$^{\mathbf{high}}$ EC percentage in healthy and psoriatic ECs.

(**A**) UMAP plot showing the expression of *IGFBP7* in healthy skin ECs within the pseudotime trajectory. The dashed line encircles *IGFBP7*^{high} ECs. *IGFBP7* expression in psoriatic ECs is provided in Figure 4C. (**B**) The proportion of *IGFBP7*^{high} ECs in healthy and psoriatic skin ECs. The light blue bars represent *IGFBP7*^{low} ECs, and the red bars represent *IGFBP7*^{high} ECs. The proportion of *IGFBP7*^{high} ECs in psoriatic skin ECs was 60.83%, whereas it was 4.11% in healthy skin ECs.

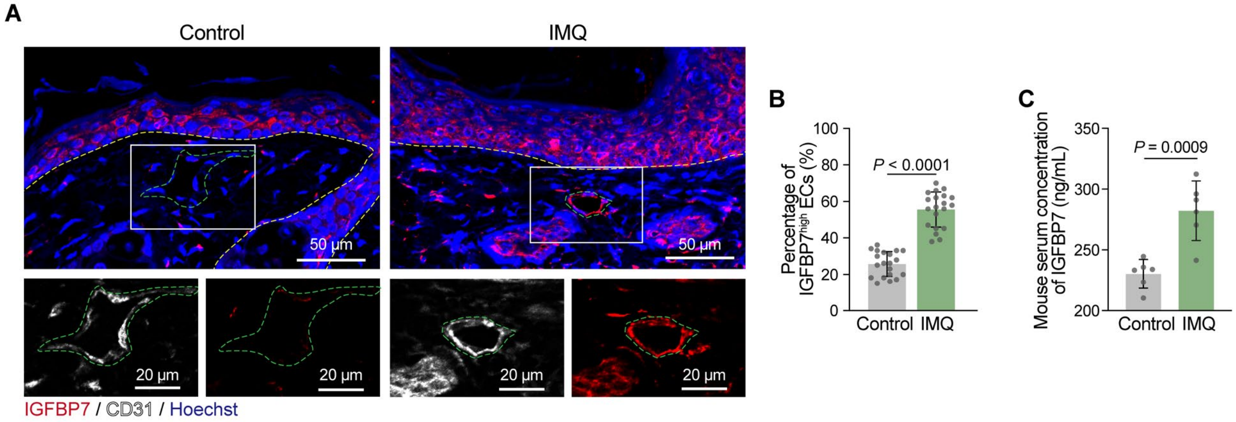

Supplemental Figure 6. The percentage of IGFBP7^{high} skin ECs in IMQ mice and control mice.

(**A**) Immunofluorescence of skin lesions from IMQ mice and normal skin from control mice (*n* = 6 mice/group) for IGFBP7 (red), CD31 (white), and Hoechst (blue). The yellow dashed line marks the interface between the epidermis and dermis. The green dashed line indicates the outline of ECs. (**B**) The percentage of IGFBP7^{high} ECs in skin blood vessels was quantified (*n* = 20 views/group). (**C**) Serum IGFBP7 levels in peripheral blood from IMQ mice and control mice (*n* = 6 mice/group). All data represent the mean ± SD. Analysis was performed using the unpaired Student's t test.

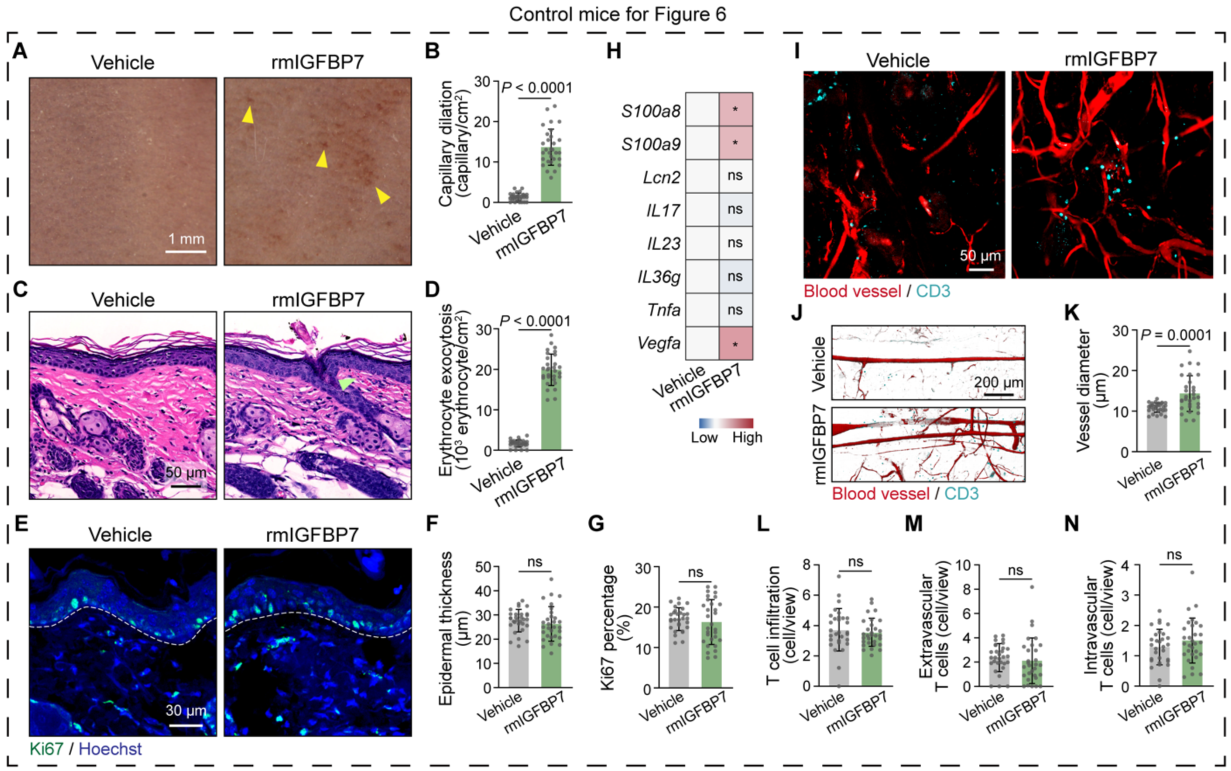

Supplemental Figure 7. IGFBP7 induces endothelial dysfunction in control mice.

(**A**) Dorsal skin appearance of control mice (n = 6 mice/group) treated with or without rmIGFBP7 under dermatoscopy. Yellow arrowheads, which point at dark red dots, indicate dilated skin capillaries. (**B**) Quantification of capillary dilation in **A**. (**C**) H&E staining of dorsal skin tissues from control mice treated with or without rmIGFBP7. Green arrowheads indicate erythrocyte exocytosis. (**D**) Quantification of erythrocyte exocytosis in **C**. (**E**) Immunofluorescence for Ki67 (green) and Hoechst (blue) in dorsal skin tissue from control mice (n = 6 mice/group) treated with or without rmIGFBP7. The dashed white line marks the interface between the epidermis and dermis. (**F** and **G**) Quantification of epidermal thickness and the percentage of Ki67^{+} cells in the basal layer in **E**. (**H**) Heatmap showing the transcriptional levels of inflammatory genes in the dorsal skin of control mice treated with or without rmIGFBP7 via qPCR. (**I**) Whole-mount immunofluorescence staining for blood vessels (red) and CD3 (cyan) in ear skin from control mice (n = 6 mice/group) treated with or without rmIGFBP7. (**J**) 3D surface rendering of blood vessels (red) and CD3 (cyan) based on whole-mount immunofluorescence staining. (**K**–**N**) The average blood vessel diameter, infiltrated T cells, extravascular T cells, and intravascular T cells were examined in **I** (n = 30 views of 6 mice/group). All data represent the mean ± SD. Analysis was performed using the unpaired Student's t test (* P<0.05).

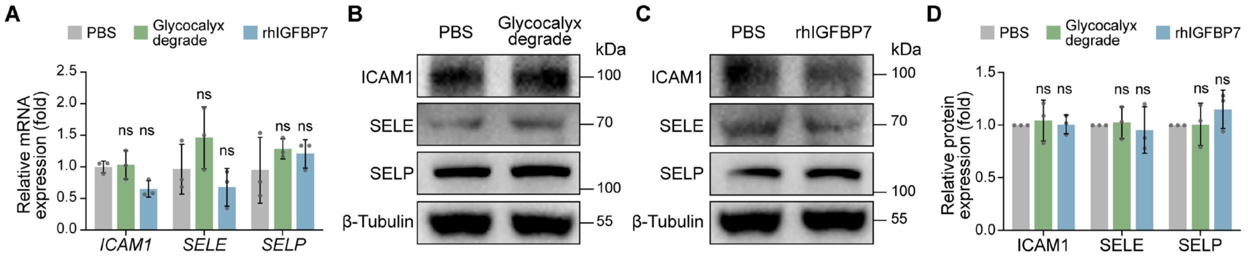

Supplemental Figure 8. The expression of adhesion molecules is not altered by endothelial glycocalyx degradation or IGFBP7.

(**A**) Relative mRNA levels of *ICAM1*, *SELE*, and *SELP* in HMEC-1 cells treated with PBS, endothelial glycocalyx degradation, or rhIGFBP7 (n = 3/group). (**B**) Representative blots of ICAM1, SELE, SELP, and β-Tubulin in HMEC-1 cells treated with PBS or endothelial glycocalyx degradation. β-Tubulin was used as the loading control (n = 3/group). (**C**) Representative blots of ICAM1, SELE, SELP, and β-Tubulin in HMEC-1 cells treated with PBS or rhIGFBP7. β-Tubulin was used as the loading control (n = 3/group). (**D**) Relative quantification of ICAM1, SELE, and SELP in HMEC-1 cells treated with PBS, endothelial glycocalyx degradation, or rhIGFBP7 in **B** and **C**. The PBS group was set as 1. All data represent the mean ± SD. Analysis was performed using the unpaired Student's t test. Labeled *P* values represent the difference between the corresponding group and the PBS group.

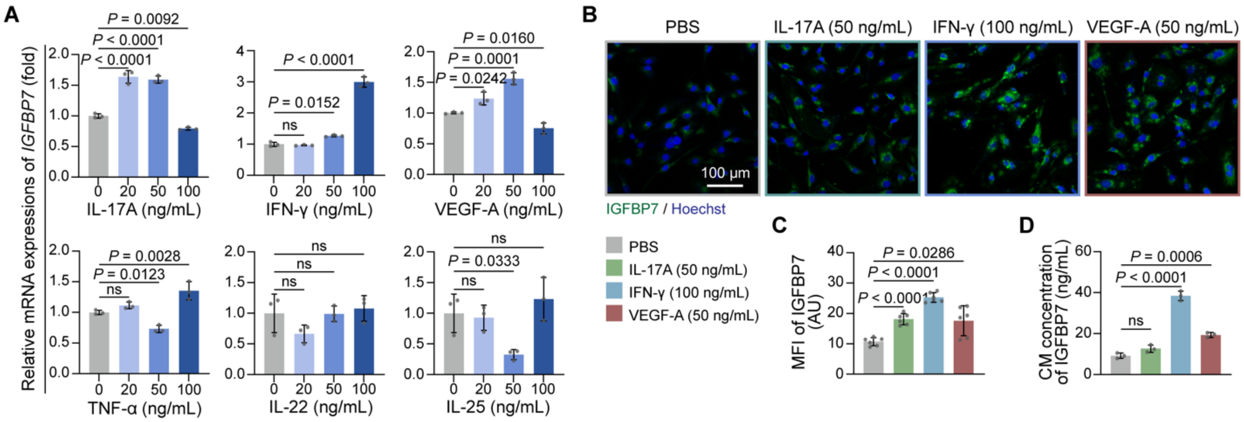

Supplemental Figure 9. IFN-γ upregulates the expression and secretion of IGFBP7 in HMEC-1 cells.

(**A**) Relative mRNA expression of *IGFBP7* in HMEC-1 cells treated with different concentrations of psoriasis-related cytokines via PCR (n = 3/group). (**B**) Immunofluorescence staining of IGFBP7 (green) and Hoechst (blue) in HMEC-1 cells (n = 6/group) treated with IL-17A (50 ng/mL), IFN-γ (100 ng/mL), and VEGF-A (50 ng/mL). (**C**) Quantification of IGFBP7 MFI in **B**. (**D**) ELISA analyzing the IGFBP7 concentration in the conditioned medium (CM) of HMEC-1 cells stimulated with IL-17A (50 ng/mL), IFN-γ (100 ng/mL), and VEGF-A (50 ng/mL) (n = 3/group). All data represent the mean ± SD. The analysis in **A** was performed using 1-way ANOVA with Dunnett's test. Analyses in **C** and **D** were performed using the unpaired Student's t test.

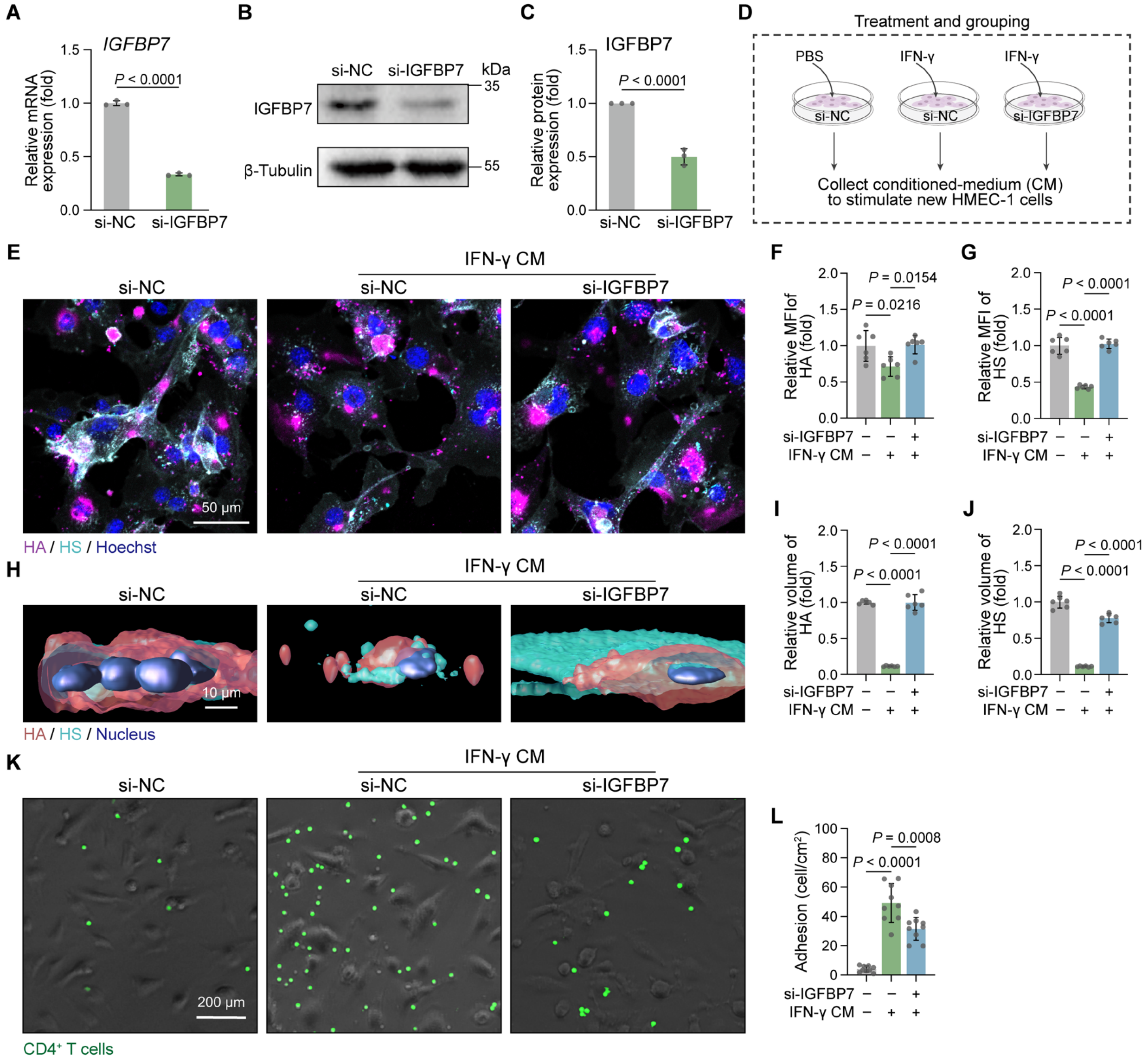

Supplemental Figure 10. Silencing IGFBP7 restores the endothelial glycocalyx and protects against T cell adhesion.

(**A**) qPCR analysis showing the relative mRNA expression of *IGFBP7* in HMEC-1 cells treated with si-IGFBP7 and si-NC (n = 3/group). The si-NC group was used as a control. (**B** and **C**) Representative blots and quantification of IGFBP7 and β-Tubulin are shown. β-Tubulin was used as the loading control (n = 3/group). The si-NC group was set as 1. (**D**) Schemes of the HMEC-1 cell grouping and treatment. Cells pretreated with si-IGFBP7 or si-NC were stimulated with IFN-γ or PBS. Then, the conditioned medium (CM) was collected as stimulation for new HMEC-1 cells in which the endothelial glycocalyx components (E–J) and adhesion capacity (K and L) were further examined. (**E**) Immunofluorescence staining of the endothelial components HA (magenta) and HS (cyan) and Hoechst (blue) in HMEC-1 cells treated with CM from the si-NC group, si-NC + IFN-γ group, and si-IGFBP7 + IFN-γ group (n = 6/group). (**F** and **G**) Relative MFIs of HA and HS were quantified in **E**. (**H**) Three-dimensional surface rendering based on immunofluorescence staining of HMEC-1 cells (n = 6/group) for HA (orange) and HS (cyan). (**I** and **J**) The relative

volume of HA and HS per cell was quantified in **H**. (**K**) Representative images of adherent $CD4^+$ T cells after 5 h of coculturing with HMEC-1 cells treated with different CMs (*n* = 9/group). (**L**) Quantification of the number of adherent $CD4^+$ T cells in **K**. All data represent the mean ± SD. Data in **A** and **C** were analyzed using the unpaired Student's t test. Data in **F**, **G**, **I**, **J**, and **L** were analyzed using 1-way ANOVA with Tukey's post hoc test.

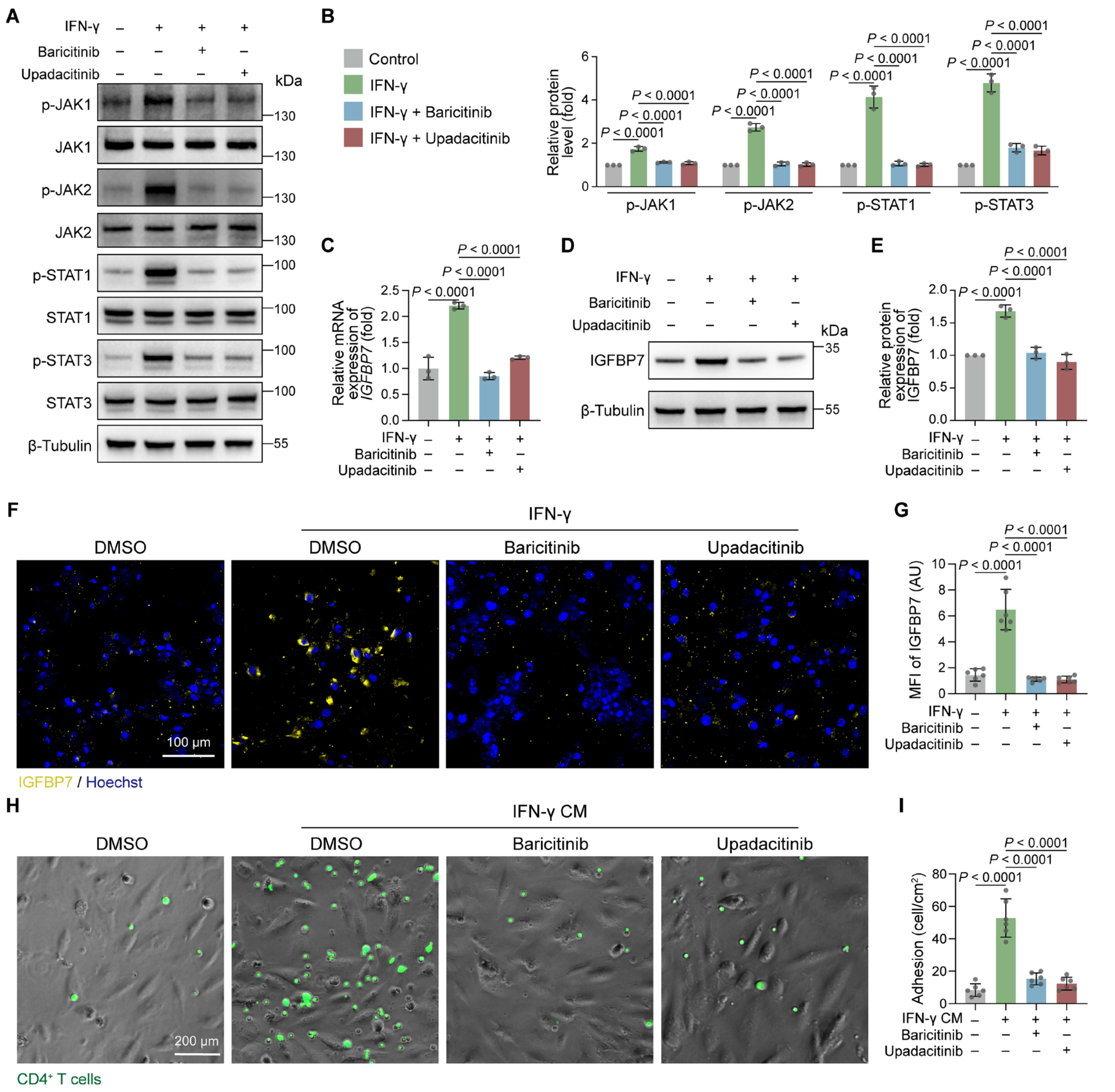

Supplemental Figure 11. IFN-γ upregulates IGFBP7 and promotes T cell adhesion via the JAK1/2-STAT1/3 pathway.

(**A** and **B**) Representative blots and quantification of JAK1, phosphorylated JAK1, JAK2, phosphorylated JAK2, STAT1, phosphorylated STAT1, STAT3, phosphorylated STAT3, and β-Tubulin in HMEC-1 cells treated with IFN-γ and JAK inhibitors (baricitinib and upadacitinib). β-Tubulin was used as the loading control (n = 3/group). The control group was set as 1. (**C**) Relative mRNA expression of *IGFBP7* in HMEC-1 cells treated with IFN-γ and JAK inhibitors (n = 3/group). (**D** and **E**) Representative blots and quantification of IGFBP7 and β-Tubulin in HMEC-1 cells treated with IFN-γ and JAK inhibitors (baricitinib and upadacitinib). β-Tubulin was used as the loading control (n = 3/group). The control group was set as 1. (**F**) Immunofluorescence staining for IGFBP7 (yellow) and Hoechst (blue) in HMEC-1 cells treated with IFN-γ and JAK inhibitors (n = 6/group). (**G**) Relative MFIs of IGFBP7 in **F** (n = 6/group). (**H**) Representative

images (*n* = 6/group) of adherent CD4$^+$ T cells after 5 h of coculturing with HMEC-1 cells treated with different conditioned media (CM). (**I**) Quantification of adherent CD4$^+$ T cells in **H** (*n* = 6/group). All data represent the mean ± SD. Analysis was performed using 1-way ANOVA with Tukey's post hoc test.

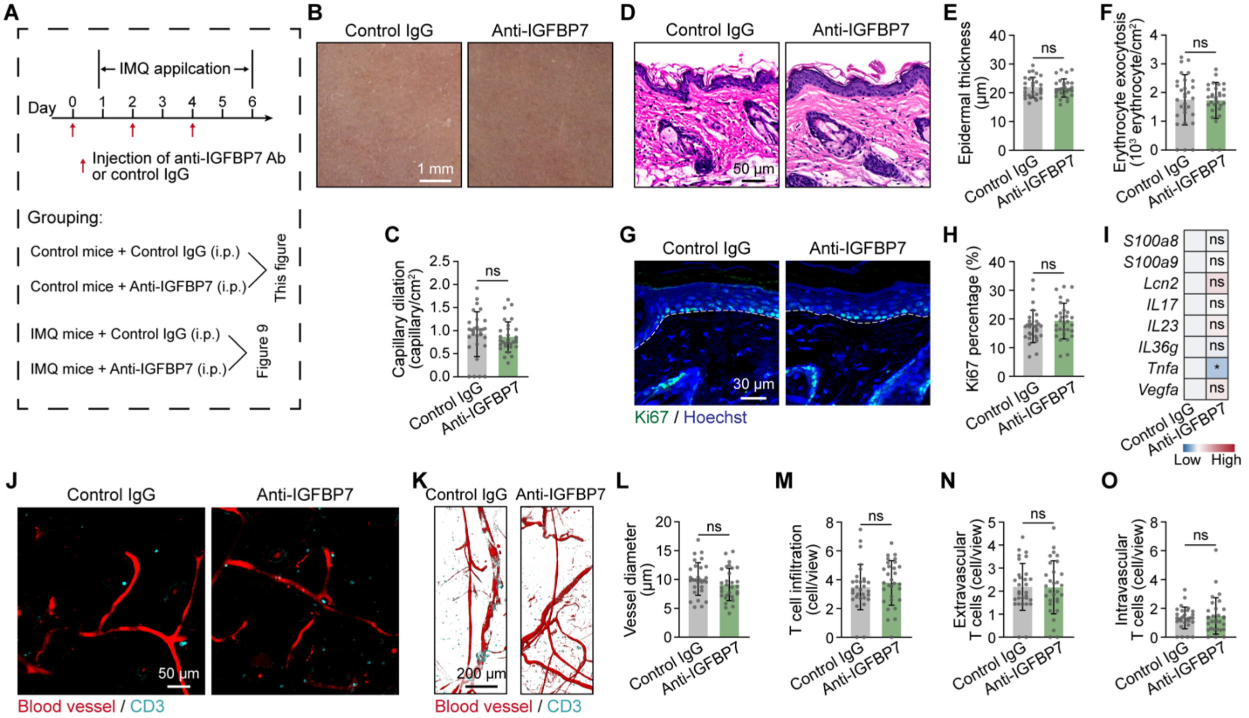

Supplemental Figure 12. Control mice with or without anti-IGFBP7 treatment.

(**A**) Scheme of the murine treatment and grouping. The first dose of anti-IGFBP7 treatment started one day before IMQ application. (**B**) Dorsal skin appearances of dilated capillaries in control mice with or without anti-IGFBP7 treatment (n = 6 mice/group). (**C**) The number of dilated capillaries in the dorsal skin was quantified in **B** (n = 30 views of 6 mice/group). (**D**) H&E staining of dorsal skin tissue in control mice with or without anti-IGFBP7 treatment (n = 6 mice/group). (**E**) Epidermal thickness of dorsal skin was quantified in **D** (n = 30 views of 6 mice/group). (**F**) Exocytotic erythrocytes were quantified in **D** (n = 30 views of 6 mice per group). (**G**) Immunofluorescence staining of dorsal skin for Ki67 (green) and Hoechst (blue) in control mice with or without anti-IGFBP7 treatment (n = 6 mice/group). (**H**) The percentage of $Ki67^+$ cells in the basal layer of the epidermis was quantified in **G**. (**I**) Heatmap showing the transcriptional levels of inflammatory genes in skin tissues via qPCR. (**J** and **K**) Whole-mount immunofluorescence staining and 3D surface rendering of blood vessels (red) and CD3 (cyan) within ear skin from control mice with or without anti-IGFBP7 treatment (n = 6 mice/group). (**L–O**) Quantifications of the average blood vessel diameter, numbers of infiltrated T cells, extravascular T cells, and intravascular T cells in **J** (n = 30 views of 6 mice/group). All data represent the mean ± SD. Analysis was performed using the unpaired Student's t test (* P<0.05).

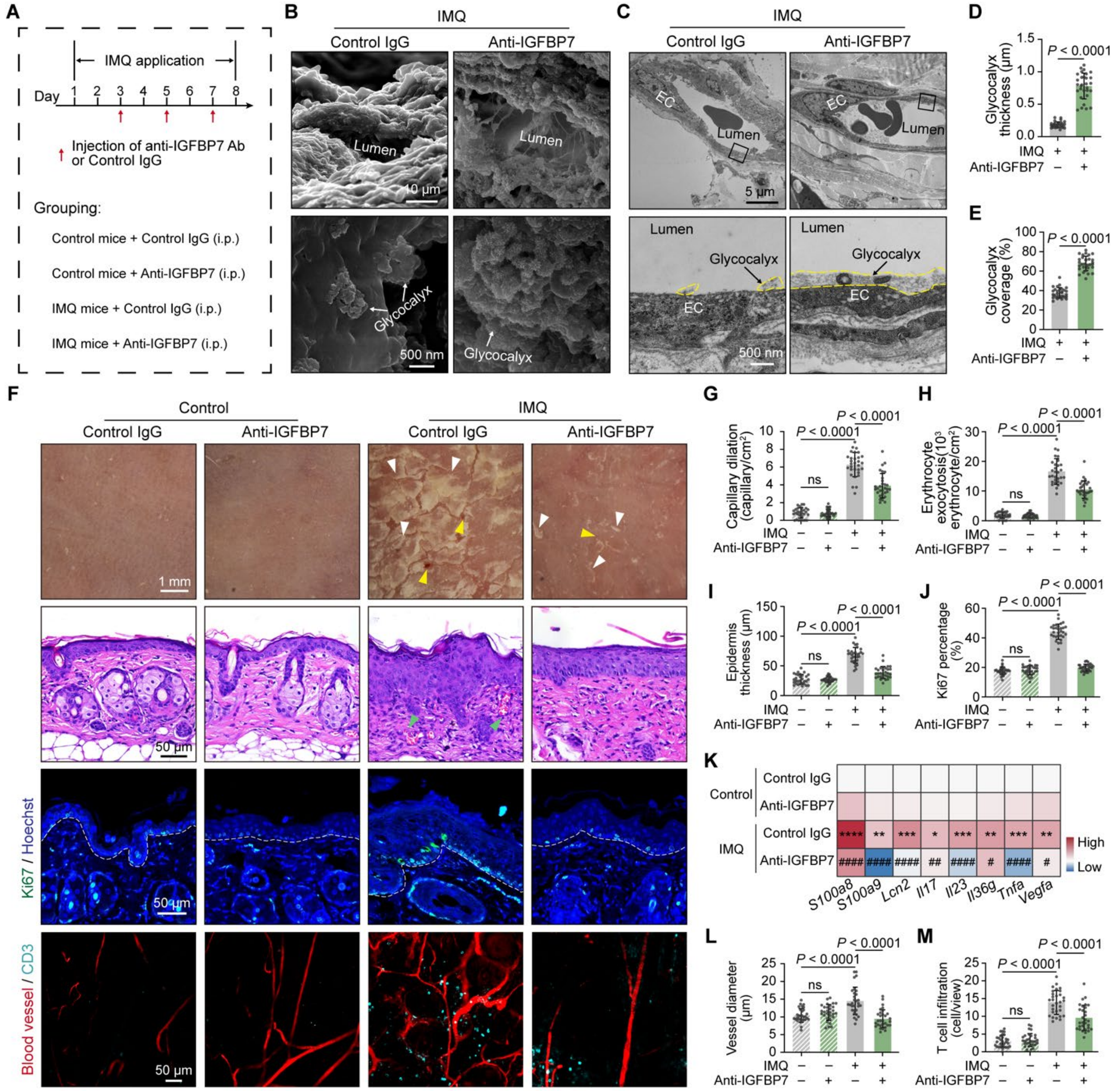

Supplemental Figure 13. Anti-IGFBP7 treatment restores the endothelial glycocalyx and reduces psoriasis-like skin inflammation in mice.

(**A**) Scheme of mouse treatments and groupings. The first dose of anti-IGFBP7 treatment started on Day 3 of IMQ application. (**B** and **C**) SEM and TEM of the endothelial glycocalyx in skin blood vessels from IMQ mice (n = 6 mice/group) with or without anti-IGFBP7 treatment. (**D** and **E**) The endothelial glycocalyx thickness and coverage (n = 30 vessels of 6 mice/group) were examined in **C**. (**F**) Dermatoscopy, H&E staining, immunofluorescence staining for Ki67 (green) and Hoechst (blue) in dorsal skin lesions, and whole-mount immunofluorescence for blood vessels (red) and CD3 (cyan) in ear skin lesions (n = 6 mice/group). Yellow arrowheads, pointing at dark red dots, indicate dilated capillaries; white arrowheads, pointing at white plates, indicate scales; green arrowheads indicate erythrocyte exocytosis; the dashed white line marks the interface between the epidermis and dermis. (**G**–**J**) Quantification of dilated capillaries (G), erythrocyte exocytosis (H),

epidermis thickness (I), and the percentage of Ki67$^+$ cells in the basal layer (J) in **F** (*n* = 30 views/group). (**K**) mRNA levels of inflammatory genes in murine skin (*n* = 6 mice/group). The asterisk represents the *P* value comparing the IMQ and control groups; the hashtag represents the *P* value comparing the IMQ after anti-IGFBP7 treatment group and IMQ after control IgG treatment group. (**L** and **M**) Blood vessel diameter and infiltrated T cells were examined in **F** (*n* = 30 views/group). All data represent the mean ± SD. Analysis of data in **D** and **E** was performed using the unpaired Student's t test. Analysis of data in **G**, **H**, **I**, **J**, **L**, and **M** was performed using 1-way ANOVA with Tukey's post hoc test. ($^{*/\#}P<0.05$, $^{**/\#\#}$ $P<0.01$, $^{***/\#\#\#}$ $P<0.001$, $^{****/\#\#\#\#}$ $P<0.0001$).

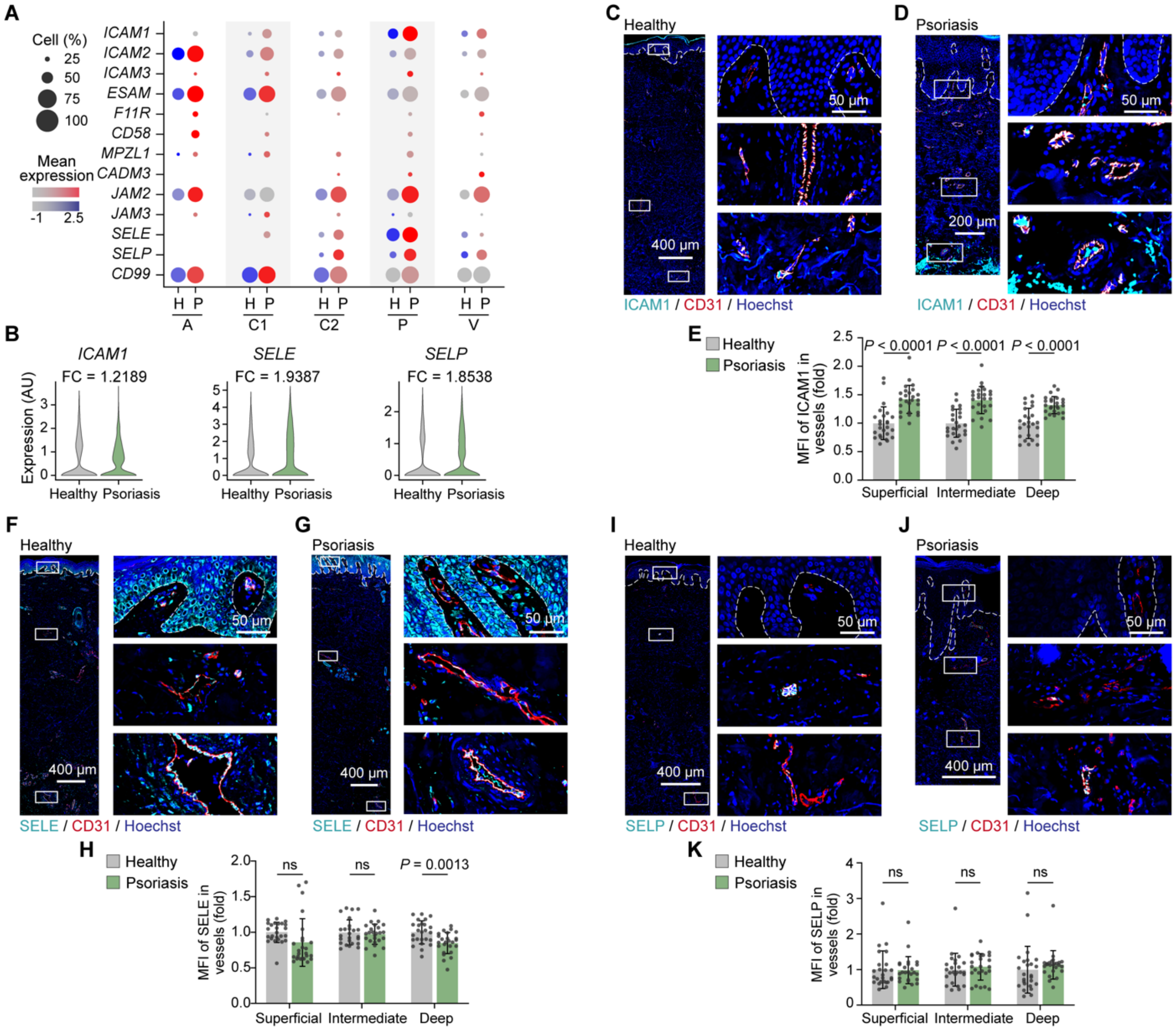

Supplemental Figure 14. Expression of adhesion molecules in skin ECs from psoriasis patients and healthy controls.

(**A**) Dot plot showing the expression of adhesion molecules in skin EC clusters of scRNA-seq datasets in healthy individuals (H) and psoriasis patients (P). The size indicates the proportion of cells expressed. Blue dots indicate expression in healthy skin ECs, and red dots indicate expression in psoriatic skin ECs. The expression level was scaled from gray to blue (healthy skin ECs) or from gray to red (psoriatic skin ECs). (**B**) Violin plots comparing the expression of *ICAM1*, *SELE*, and *SELP* in ECs from psoriatic and healthy skin. Fold change (FC) of gene expression in psoriasis ECs vs. healthy ECs was shown. (**C**–**K**) Immunofluorescence of ICAM1, SELE, SELP, and CD31 in psoriatic and healthy skin (n = 7 skin samples/group). Images of the superficial, intermediate, and deep plexuses are magnified. The white dotted line marks the interface between the epidermis and dermis. MFIs of ICAM1, SELE, and SELP in the CD31-positive area were compared between healthy and psoriatic skin (n = 25 vessels in each plexus/group). All data represent the mean ± SD. Analysis was performed using the unpaired Student's t test.

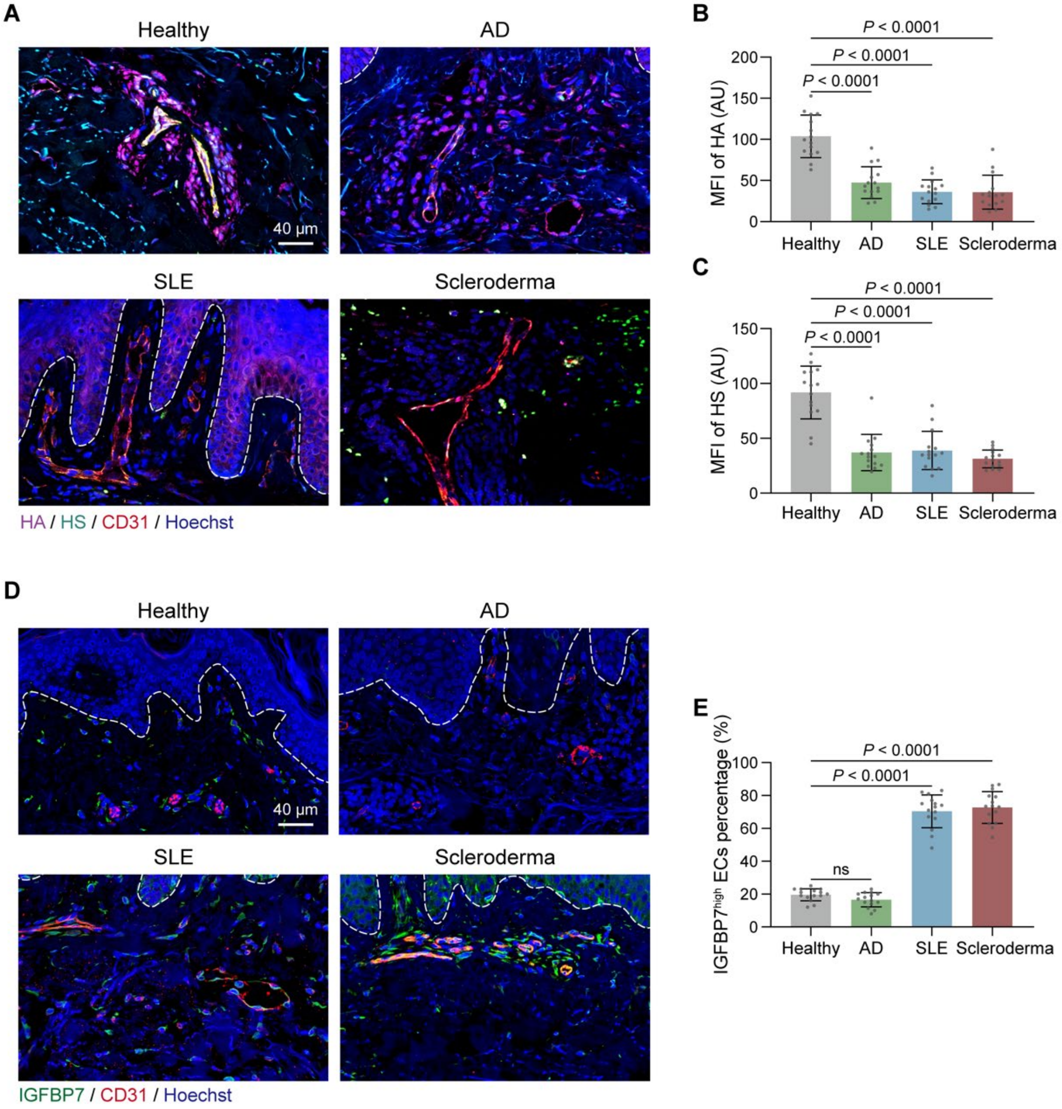

Supplemental Figure 15. Endothelial glycocalyx and IGFBP7 expression in different inflammatory dermatoses.

(**A**) Immunofluorescence of the endothelial glycocalyx components HA (magenta) and HS (cyan), CD31 (red), and Hoechst (blue) in skin tissues from healthy individuals, AD, SLE, and scleroderma patients (*n* = 3 skin samples/group). (**B** and **C**) Quantification of the MFI of HA and HS in **A** (*n* = 15 vessels of 3 skin samples/group). (**D**) Immunofluorescence of IGFBP7 (cyan), CD31 (red), and Hoechst (blue) in skin tissues from healthy individuals, AD, SLE, and scleroderma patients (*n* = 3 skin samples/group). (**E**) The percentage of $IGFBP7^{high}$ ECs in blood vessels was quantified in **D** (*n* = 15 vessels of 3 skin samples/group). All data represent the mean ± SD. Analysis was performed using the unpaired Student's t test.

Supplemental Table 1.

Clinical information of healthy donors and patients with psoriasis vulgaris, AD, SLE, and scleroderma.

No.	Donor	Age	Gender	PASI	Experiment
H 1	healthy	33	male	/	immunofluorescence staining
H 2	healthy	24	female	/	immunofluorescence staining
H 3	healthy	30	female	/	immunofluorescence staining
H 4	healthy	31	male	/	immunofluorescence staining
H 5	healthy	43	female	/	immunofluorescence staining
H 6	healthy	37	female	/	immunofluorescence staining
H 7	healthy	25	female	/	immunofluorescence staining
H 8	healthy	23	male	/	TEM/SEM
H 9	healthy	29	female	/	TEM/SEM
H 10	healthy	32	male	/	TEM/SEM
H 11	healthy	22	male	/	ELISA
H 12	healthy	23	male	/	ELISA
H 13	healthy	24	male	/	ELISA
H 14	healthy	24	male	/	ELISA
H 15	healthy	27	male	/	ELISA
H 16	healthy	28	male	/	ELISA
H 17	healthy	30	male	/	ELISA
H 18	healthy	31	male	/	ELISA
H 19	healthy	32	male	/	ELISA
H 20	healthy	33	male	/	ELISA
H 21	healthy	35	male	/	ELISA
H 22	healthy	36	male	/	ELISA
H 23	healthy	36	male	/	ELISA
H 24	healthy	43	male	/	ELISA
H 25	healthy	45	male	/	ELISA
H 26	healthy	22	female	/	ELISA
H 27	healthy	24	female	/	ELISA
H 28	healthy	24	female	/	ELISA
H 29	healthy	25	female	/	ELISA
H 30	healthy	28	female	/	ELISA
H 31	healthy	30	female	/	ELISA
H 32	healthy	31	female	/	ELISA
H 33	healthy	32	female	/	ELISA
H 34	healthy	33	female	/	ELISA
H 35	healthy	35	female	/	ELISA
H 36	healthy	37	female	/	ELISA
H 37	healthy	39	female	/	ELISA

PV 1	psoriasis vulgaris	20	female	10.2	scRNA-seq
PV 2	psoriasis vulgaris	25	male	11.5	scRNA-seq
PV 3	psoriasis vulgaris	37	female	16.4	scRNA-seq
PV 4	psoriasis vulgaris	60	female	21.6	scRNA-seq
PV 5	psoriasis vulgaris	34	female	11	immunofluorescence staining
PV 6	psoriasis vulgaris	30	male	16.9	immunofluorescence staining
PV 7	psoriasis vulgaris	47	male	28.2	immunofluorescence staining
PV 8	psoriasis vulgaris	45	male	14	immunofluorescence staining
PV 9	psoriasis vulgaris	39	female	28.8	immunofluorescence staining
PV 10	psoriasis vulgaris	18	male	12	immunofluorescence staining
PV 11	psoriasis vulgaris	32	male	10.5	immunofluorescence staining
PV 12	psoriasis vulgaris	42	female	15.4	TEM/SEM
PV 13	psoriasis vulgaris	28	male	10.6	TEM/SEM
PV 14	psoriasis vulgaris	37	male	18.4	TEM/SEM
PV 15	psoriasis vulgaris	24	male	2.3	ELISA
PV 16	psoriasis vulgaris	32	male	2.3	ELISA
PV 17	psoriasis vulgaris	24	female	2.4	ELISA
PV 18	psoriasis vulgaris	25	female	2.4	ELISA
PV 19	psoriasis vulgaris	30	female	3	ELISA
PV 20	psoriasis vulgaris	45	female	5.6	ELISA
PV 21	psoriasis vulgaris	33	male	6.3	ELISA
PV 22	psoriasis vulgaris	32	female	6.5	ELISA
PV 23	psoriasis vulgaris	41	female	6.8	ELISA
PV 24	psoriasis vulgaris	28	male	7.5	ELISA
PV 25	psoriasis vulgaris	48	female	7.7	ELISA
PV 26	psoriasis vulgaris	23	male	8.2	ELISA
PV 27	psoriasis vulgaris	45	female	10.9	ELISA
PV 28	psoriasis vulgaris	22	male	11.2	ELISA
PV 29	psoriasis vulgaris	28	female	13.2	ELISA
PV 30	psoriasis vulgaris	43	male	13.8	ELISA
PV 31	psoriasis vulgaris	18	male	14	ELISA
PV 32	psoriasis vulgaris	18	male	14.4	ELISA
PV 33	psoriasis vulgaris	44	male	15.2	ELISA
PV 34	psoriasis vulgaris	47	male	19	ELISA
PV 35	psoriasis vulgaris	36	male	19.2	ELISA
PV 36	psoriasis vulgaris	45	male	19.6	ELISA
PV 37	psoriasis vulgaris	27	male	19.8	ELISA
PV 38	psoriasis vulgaris	36	male	22.2	ELISA
PV 39	psoriasis vulgaris	57	female	22.2	ELISA
PV 40	psoriasis vulgaris	39	female	23.4	ELISA
PV 41	psoriasis vulgaris	33	female	24.3	ELISA

PV 42	psoriasis vulgaris	35	male	25.6	ELISA
PV 43	psoriasis vulgaris	30	male	25.8	ELISA
PV 44	psoriasis vulgaris	31	male	29.4	ELISA
PV 45	psoriasis vulgaris	20	male	32	ELISA
PV 46	psoriasis vulgaris	31	female	33	ELISA
AD 1	atopic dermatitis	4	female	/	immunofluorescence staining
AD 2	atopic dermatitis	8	female	/	immunofluorescence staining
AD 3	atopic dermatitis	10	male	/	immunofluorescence staining
SLE 1	systemic lupus erythematosus	26	female	/	immunofluorescence staining
SLE 2	systemic lupus erythematosus	23	male	/	immunofluorescence staining
SLE 3	systemic lupus erythematosus	33	male	/	immunofluorescence staining
Scl 1	scleroderma	22	male	/	immunofluorescence staining
Scl 2	scleroderma	30	male	/	immunofluorescence staining
Scl 3	scleroderma	45	female	/	immunofluorescence staining

PASI: psoriasis area and severity index.

Supplemental Table 2.

Primary antibody information.

Target	Supplier	Catalog	Usage	Dilution
CD31	Abcam	ab199012	immunofluorescence staining	1:400
HS	Abcam	ab2501	immunofluorescence staining	1:200
HA	Abcam	ab53842	immunofluorescence staining	1:200
CD3	Abcam	ab135372	immunofluorescence staining	1:200
IGFBP7	Abcam	ab74169	immunofluorescence staining/ western blotting	1:1000/ 1:1000
Ki67	Abcam	ab15580	immunofluorescence staining	1:200
ICAM1	Abcam	ab109361	immunofluorescence staining/ western blotting	1:400/ 1:1000
SELE	ABclonal	A2191	immunofluorescence staining/ western blotting	1:200/ 1:1000
SELP	Abcam	ab178424	immunofluorescence staining/ western blotting	1:200/ 1:1000
JAK1	Proteintech	66466-1-lg	western blotting	1:1000
phospho-JAK1	ABclonal	AP0530	western blotting	1:1000
JAK2	ABclonal	A19629	western blotting	1:1000
phospho-JAK2	ABclonal	AP0531	western blotting	1:1000
STAT1	Cell Signaling Technology	14994	western blotting	1:1000
phospho-STAT1	Cell Signaling Technology	7649	western blotting	1:1000
STAT3	Cell Signaling Technology	9139	western blotting	1:1000
phospho-STAT3	Cell Signaling Technology	9145	western blotting	1:1000
β-Tubulin	Immunoway	YT4780	western blotting	1:2000

Supplemental Table 3.

Primer sequences for qPCR.

Primers	Species	Forward (5’-3’)	Reverse (5’-3’)
IGFBP7	Homo sapiens	tgaagtaactggctgggtgc	tagctcggcaccttcacctt
SELE	Homo sapiens	ccgagcgaggctacatgaat	gcatcgcatctcacagcttc
SELP	Homo sapiens	gcggtggcttctacgatagg	ttcatgggtgtttatggaaacctta
ICAM1	Homo sapiens	tgaccatctacagctttccg	tggaaccccattcagcgtc
18S	Homo sapiens	cagccacccgagattgagca	tagtagcgacgggcggtgtg
S100a8	Mus musculus	aaatcaccatgccctctacaag	cccacttttatcaccatcgcaa
S100a9	Mus musculus	ggaaggaaggacaccctgac	ggcttcatttctcttctctttcttc
Lcn2	Mus musculus	tctgtccccaccgaccaa	ggaaagatggagtggcagaca
Il17a	Mus musculus	gagagcttcatctgtgtctctg	gcgccaagggagttaaagac
Il23	Mus musculus	accagcgggacatatgaatc	agtccttgtgggtcacaacc
Il36g	Mus musculus	cacagagtaaccccagtcagc	ttcattggctcagggtggtg
Tnfa	Mus musculus	ggtccccaaagggatgagaagt	ttgctacgacgtgggctaca
Vegfa	Mus musculus	tattcagcggactcaccagc	aaccaacctcctcaaaccgt
Actb	Mus musculus	aacagtccgcctagaagcac	cgttgacatccgtaaagacc

Supplemental Movie 1.

T cells rolling on control HMEC-1 cells.

Supplemental Movie 2.

T cells rolling on HMEC-1 cells with degraded endothelial glycocalyx.

Supplemental Movie 3.

T cells rolling on HMEC-1 cells pretreated with rhIGFBP7.

Supplemental Data 1. (Excel file)

Top 50 differentially expressed genes in 5 EC clusters from healthy and psoriatic skin. The column avg_logFC represents the average log [normalized expression of the current group of cells/normalized expression of the rest of the cells]. The column pct.1 represents the percentage of cells expressed in the current group of cells. The column pct.2 represents the percentage of cells expressed in the rest of the cells.

Supplemental Data 2. (Excel file)

Top 50 differentially expressed genes in IGFBP7^{high} psoriatic skin ECs and IGFBP7^{low} psoriatic skin ECs. The column norm_total_mean_high or norm_total_mean_low represents the normalized total mean of counts in IGFBP7^{high} or IGFBP7^{low} psoriatic ECs. Norm_foldchange represents [norm_total_mean_high/norm_total_mean_low]. The chi2LR1 represents the Chi-square statistic for hypothesis testing of H0. The column pvalue.adj.FDR represents adjusted *P* value of the H0 *P* value using Benjamini & Hochberg's method.
